# *Fallopia Japonica* and *Prunella vulgaris* inhibit myopia progression by suppressing AKT and NFκB mediated inflammatory reactions

**DOI:** 10.1186/s12906-022-03747-2

**Published:** 2022-10-14

**Authors:** Chih-Sheng Chen, Yu-An Hsu, Chia-Hung Lin, Yao-Chien Wang, En-Shyh Lin, Ching-Yao Chang, Jamie Jiin-Yi Chen, Ming-Yen Wu, Hui-Ju Lin, Lei Wan

**Affiliations:** 1 Department of Food Nutrition and Health Biotechnology, Asia University, Taichung, Taiwan; 2 Division of Chinese Medicine, Asia University Hospital, Taichung, Taiwan; 3 School of Chinese Medicine, China Medical University, 91, Hsueh-Shih Road, Taichung, 40402 Taiwan; 4 Department of Emergency Medicine, Taichung Tzu Chi Hospital, Taichung, Taiwan; 5 Department of Beauty Science, National Taichung University of Science and Technology, Taichung, Taiwan; 6 Department of Medical Laboratory Science and Biotechnology, Asia University, Taichung, Taiwan; 7 Eye Center, China Medical University Hospital, Taichung, Taiwan; 8 Department of Obstetrics and Gynecology, China Medical University Hospital, Taichung, Taiwan

**Keywords:** Myopia, Monocular form deprivation (MFD), Inflammation, *Fallopia Japonica* (FJ), *Prunella Vulgaris* (PV)

## Abstract

**Background:**

The increased global incidence of myopia requires the establishment of therapeutic approaches. This study aimed to investigate the effect of *Fallopia Japonica* (FJ) and *Prunella vulgaris* (PV) extract on myopia caused by monocular form deprivation (MFD).

**Methods:**

We used human retinal pigment epithelial cell to study the molecular mechanisms on how FJ extract (FJE) and PV extract (PVE) lowering the inflammation of the eye. The effect of FJE and PVE in MFD induced hamster model and explore the role of inflammation cytokines in myopia.

**Results:**

FJE + PVE reduced IL-6, IL-8, and TNF-α expression in RPE cells. Furthermore, FJE and PVE inhibited inflammation by attenuating the phosphorylation of protein kinase B (AKT), and nuclear factor kappa-light-chain-enhancer of activated B (NF-κB) pathway. In addition, we report two resveratrol + ursolic acid compounds from FJ and PV and their inhibitory activities against IL-6, IL-8, and TNF-α expression levels in RPE cells treated with IL-6 and TNF-α. FJE, PVE, and FJE + PVE were applied to MFD hamsters and their axial length was measured after 21 days. The axial length showed statistically significant differences between phosphate-buffered saline- and FJE-, PVE-, and FJE + PVE-treated MFD eyes. FJE + PVE suppressed expressions of IL-6, IL-8, and TNF-α. They also inhibited myopia-related transforming growth factor-beta (TGF)-β1, matrix metalloproteinase (MMP)-2, and NF-κB expression while increasing type I collagen expression.

**Conclusions:**

Overall, these results suggest that FJE + PVE may have a therapeutic effect on myopia and be used as a potential treatment option.

**Supplementary Information:**

The online version contains supplementary material available at 10.1186/s12906-022-03747-2.

## Background

Myopia is one of the most common refractive disorders in humans and its prevalence has been increasing over the past decades. By 2050, it is expected that approximately 4.8 billion people will be myopic [1, 2]. Most of the patients with myopia have the excessive elongation of the vitreous chamber, which largely accounts for the increase in eye elongation, loss of scleral tissue, and degenerative changes, such as atrophy of the retina and choroid [3]. Myopia is also a major risk factor for severe sight-threatening pathologies including cataract, glaucoma, choroidal neovascularization, and macular and retinal complications [2, 4].

Although the underlying molecular mechanisms of myopia progression are not fully understood, accumulated evidence has demonstrated that inflammation plays an important role in the pathogenesis of myopia [4–7]. Several studies indicated a role for inflammation in myopia progression and increased prevalence of myopia in children with an inflammatory disease, such as type 1 and 2 diabetes, systemic lupus erythematosus, uveitis, allergic diseases, and Kawasaki disease [4–6]. Our recent animal model showed that inflammatory markers, such as interleukin (IL)-6, IL-8, tumor necrosis factor (TNF)-α, transforming growth factor-beta (TGF)-β, and nuclear factor kappa-light-chain-enhancer of activated B (NF-κB) are upregulated in myopic eyes. Increasing myopia prevalence and subsequent consequences pose a major public health concern. Although spectacle correction can improve vision, myopia is associated with an increased risk of retinal detachment, myopic macular degeneration, cataract, and glaucoma. There are currently no pharmaceutical agents approved by the FDA in the US for use in myopia treatment, although researchers and clinicians are searching for better therapies.

Plants provide an abundant source of primary compounds for a variety of diseases. *Fallopia Japonica* (FJ) and *Prunella vulgaris* (PV) are used in traditional Chinese medicine for the treatment of various inflammatory diseases and are widely distributed in China, Kapan, Korea, and Europe [8–11]. FJ and PV belong to the group of polyphenols. Main constituents of FJ include resveratrol, polydatin, emodin, physcion, chrysophanol, and rhein [9, 12]. Resveratrol is a naturally occurring anti-inflammatory compound typically associated with red wine and is also present in FJ [12]. PV is a perennial herb also known as the self-heal herb and is a standard medicinal material in the Chinese Pharmacopoeia. Ursolic acid, a pentacyclic triterpene acid, is found in PV, which exhibits many bioactivities, including anti-inflammation, anti-hyperglycemia, and anti-tumor effects [13, 14].

A variety of animals, including tree shrew, monkey, chicken, guinea pig, rat, mice, and Syrian hamster have been used to investigate the mechanisms of myopia progression [4]. The main methods for inducing myopia in mice are monocular form deprivation myopia (MFD) and lens induced myopia (LIM) [15]. MFD and LIM are two different types of experimental myopia. MFD is through prohibiting animals to see whereas LIM is through wearing concave lens before an animal’s eyes to effect image formation behind the retina [16]. Both MFD [4] and LIM[17] animal models revealed that inflammation is involved in the development of myopia. We hypothesize that FP and PV extracts can improve myopia progression via decreasing inflammation. This study was designed to investigate the effect of FJ and PV in MFD-induced hamster model and explore the role of inflammatory cytokines in myopia. We demonstrated that FJ and PV extracts may attenuate myopia progression via inhibiting inflammation. Our study provides insight into myopia and supports the potential therapeutic value of FJ and PV in myopia treatment.

## Materials and methods

### Cell culture

The ARPE-19 cells were purchased from the Bioresource Collection and Research Center, Hsinchu, Taiwan (BCRC; BCRC-60, 383). The ARPE-19 cells were cultured in Dulbecco’ Modified Eagle’s medium (DMEM) (Cat# 12,100,046, Gibco, Thermo Fisher SCIENTIFIC, MA, USA) containing sodium bicarbonate, 10% fetal bovine serum (FBS) (Cat# 16,000,044, Gibco, Thermo Fisher SCIENTIFIC, MA, USA) and 1% penicillin–Streptomycin (PS) (Cat# 15,140,122, Gibco, Thermo Fisher SCIENTIFIC, MA, USA), at 37 °C in a 5% CO_2_ incubator, with the medium being replaced every 2–3 days. Human RPE (H-RPE) cells were obtained from the Lonza (Cat# 00,194,987, Nj, USA). The H-RPE cells were cultured in Retinal Pigment Epithelial Cell Growth Medium BulletKit (Cat# 00,195,409, Nj, USA), at 37 °C in a 5% CO_2_ incubator, with the medium being replaced every 4–5 days.

### Sample preparation and extraction

FJ and PV were purchased from Herbal Market (Taichung, Taiwan). Water Extraction: 100 g of dried FJ and PV sample was extracted with 500 mL boiling water. The plant material was steeped with stirring for 1 h, and then centrifuged at 3000 rpm at 4℃ to give a clear supernatant and filtered through Whatman Number 4 filter paper. Crude extract filtrates (FJE and PVE) were successively filtered using 0.45 and 0.2-µm Acrodisc syringe filters (Pall Life Sciences, USA). The filtered sample was frozen for 12 h in a deep freezer at − 80 °C. The frozen extract was lyophilized for 4 or 5 days in a freeze dryer (CoolSafe Freeze Dryers, LaboGene Co., Bjarkesvej, Denmark) at − 45 to − 55 °C. The dry sample concentrations are 4.5 and 3 mg/ml of FJE and PVE. We reconstituted the dry extract with distilled water for cell model and animal model. Resveratrol (purity, 99%) and ursolic acid (purity, 99%) were purchased from Sigma (St. Louis, MO, USA) and were dissolved in dimethylsulfoxide (DMSO).

### Cell viability assay

Cell viability was determined using the MTS/PMS ((3-(4,5-dimethylthiazol-2-yl)-5-(3-carboxymethoxyphenyl)-2(4-sulfophenyl)-2H-tetrazolium, inner salt)/phenazine methosulfate) assay (Cat# G5421, Promega, WI, USA). ARPE-19 cells were seeded in 96 well plates (3 × 10^3^ cells/well). Media containing different concentrations (0, 10, 20, 30, 40, 60, 80, 100 and 120 μg/ml) of the FJE and PVE were added and incubated for 72 h. In addition, different dilutions of resveratrol, ursolic acid, and resveratrol + ursolic acid were added and incubated further for 72 h. Herein, 20 μl of MTS was subsequently added from a stock solution (2 mg/mL) and incubated for an additional 2 h. The absorbance was read at 490 nm using the microplate reader 550 model (Bio-rad).

### ELISA immunoassay

Cytokines were detected in the supernatants of ARPE-19 and H-RPE cells, seeded at 10,000 cells/well in 96-well plates. ARPE-19 cells were pretreated with 5 ng/ml of different kinds of cytokines IL-6 (Cat# 200–06, PeproTech, NJ, USA), (TNF-α (Cat# 300-01A, PeproTech, NJ, USA), IL-6 + TNF-α for 16 h. Cell-free supernatants were collected and stored at -80 °C until further use. ARPE-19 and H-RPE cells pretreated with 5 ng/ml of cytokines IL-6 + TNF-α for 2 h. Treatment media were subsequently removed and fresh media with or without treatment were applied and incubated for 6 h. Cell-free supernatants were collected and stored at -80 °C until further use. Levels of IL-6, IL-8, and TNF-α were determined using a human IL-6 (Cat# 88–7066-22, Thermo Fisher SCIENTIFIC, MA, USA), IL-8 (Cat# 88–8086-22, Thermo Fisher SCIENTIFIC, MA, USA), and TNF-α (Cat# 88–7399-22, Thermo Fisher SCIENTIFIC, MA, USA) ELISA Ready-Set-Go kit following the manufacturer’s instructions.

### Western blot analysis

Cells were lysed in RIPA lysis buffer (10 mM Tris–Cl, 100 mM NaCl, 1 mM EDTA, 1 mM EGTA, 1 mM NaF, 20 mM Na_4_P_2_O_7_, 2 mM Na_3_VO_4_, 1% Triton X-100, 10% glycerol, 0.1% sodium dodecyl sulfate, and 0.5% deoxycholate) containing protease inhibitors (Roche Applied Science, Madison, USA) and phosphatase inhibitors (Roche Applied Science, USA) on ice for 30 min. After centrifugation at 4 °C for 30 min (12,000 rpm), the supernatant was collected. Samples (15 μg protein) were loaded on sodium dodecyl sulfate–polyacrylamide gel (SDS-PAGE). The primary antibodies used included AKT (Cat# 9272, Cell signaling Technology, MA, USA), phosphor-AKT (Ser473) (Cat# 4060, Cell signaling Technology, MA, USA), NF-κB (Cat# 3034, Cell signaling Technology, MA, USA), and phosphor-NF-κB (p65, Ser536) (Cat# 3031, Cell signaling Technology, MA, USA) and β-actin (Cat# ab8227, Abcam, Cambridge, UK). The primary antibodies were diluted 1:1000 in PBS-5% milk. The membranes were detection was performed with enhanced chemiluminescence kit (ECL, Pierce, Thermo Fisher SCIENTIFIC, MA, USA) and an ImageQuant LAS-4000 Chemiluminescence and Fluorescence Imaging System (GE Healthcare, Illinois, USA).

### Animals

Experimental animals: All animal experiments in this study were approved by the Institutional Animal Care and Use Committee of China Medical University (approval number: 2017–298-1) and were in accordance with the guidelines for the Use of Animals in Ophthalmic and Vision Research and ARRIVE guidelines. All animal experiment was performed in the Laboratory Animal Center of China Medical University. We purchased male Golden Syrian hamsters (three-week-old) from National Laboratory Animal Center (Taiwan). The hamsters were maintained in a specific pathogen-free animal facility at China Medical University. The animals were kept under a 12 h light/12 h dark cycle. For experiments on hamsters' behavior, the intake of water and food was not limited. We used a previously established hamster model of myopia by MFD with right eyelid fusion for 21 days [4]. The right eyes were sutured with 6–0 PROLENE nonabsorbable sutures, blue monofilament (W8706, ETHICON, USA) on day 21 after birth. The left eyes were left open and were served as contralateral control eyes. The hamsters were randomly separated into four groups (*n* = 10 animals each), each group receiving a different topical treatment. The three groups were: (1) control (hamsters received balanced salt solution (BSS)); (2) 150 ng/ml FJE (3) 150 ng/ml PVE (4) 150 ng/ml FJE + PVE. Hamsters were raised with a right eyelid fusion for 21 days. The indicated treatments were administered at a volume of 10 μl, which were applied topically to both eyes of the hamsters twice a day (8 AM, 5 PM) until they were euthanized. All animals were sacrificed in a CO_2_ chamber. Before the animals were anesthetized by CO_2_ gas and sacrificed, the axial lengths of the hamsters were measured. The axial length of the eye was defined as the distance from the front of the cornea to the back of the sclera. The axial lengths of each left and right eye were measured by A-scan ultrasonography (PacScan Plus, New Hyde Park NY, USA), and the axial lengths of three independent measurements were averaged.

### Immunohistochemistry (IHC)

Eyes were collected from the control, FJE, PVE, and FJE + PVE, fixed overnight in 4% paraformaldehyde in phosphate buffer and embedded in paraffin. Eyes tissue blocks were sectioned with an 8 μm thickness and mounted on clean glass slides. The slides were exposed to a PBS solution of 5% normal goat serum, blocked for 1 h at room temperature, and then incubated overnight at 4 °C with the specific primary antibody IL-6 (1:500, Cat# ab6672, Abcam, Cambridge, UK), IL-8 (1:200, Cat# MBS551025, MyBioSource, CA, USA), TNF-α (1:500, Cat# BS1857, Bioworld, TX, USA), TGF-β1 (1:100, Cat# ab66043, Abcam, Cambridge, UK), MMP-2 (1:500, Cat# ab37150, Abcam, Cambridge, UK), type I collagen (1:100, Cat# GTX20292, GeneTex, Hsinchu, TAIWAN), and NF-κB (1 μg/ml Cat# ab16502, Abcam, MA, USA).

### Software and statistical analysis

Each result was expressed as mean ± standard deviation (SD). Unpaired independent t-test and one-way ANOVA analysis of variance were performed to compare the differences between the two groups using the GraphPad prism software. A *P*-value < 0.05 was considered to be significant.

## Results

### Effect of FJE- and PVE-treated ARPE-19 cell viabilit*y*

**Fig. 1:**
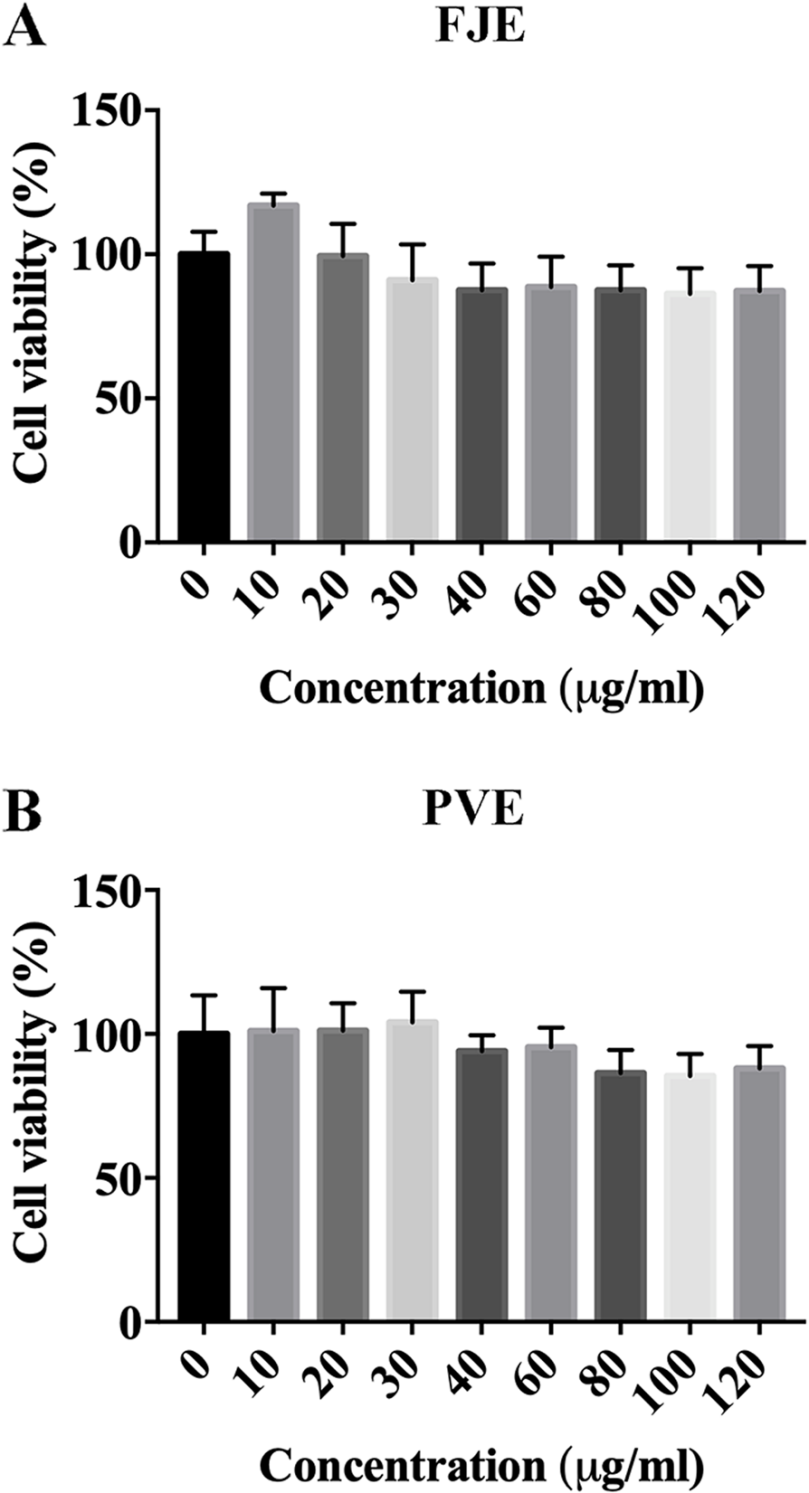
Viability of ARPE-19 cells treated with different concentration of FJE and PVE. **A** and **B** Different concentrations of FJE and PVE (0, 10, 20, 30, 40, 60, 80, 100, and 120 μg/ml) were applied for 72 h. Cell viability was determined using MTS assay. The data are expressed as the mean  ± SD of three independent experiments

To evaluate the cytotoxicity of FJE and PVE on ARPE-19 cells, the cells were treated with a series of concentrations of FJE and PVE for 72 h (0, 10, 20, 30, 40, 60, 80, 100, and 120 μg/ml). Cell viability is as shown in Fig. 1A, and B, FJE (10–120 μg) and PVE (10–120 μg) no cytotoxicity in ARPE-19 cells.

### Pro-inflammatory cytokines induce the expression of IL-6, IL-8, and TNF-α in ARPE-19 cells

**Fig. 2:**
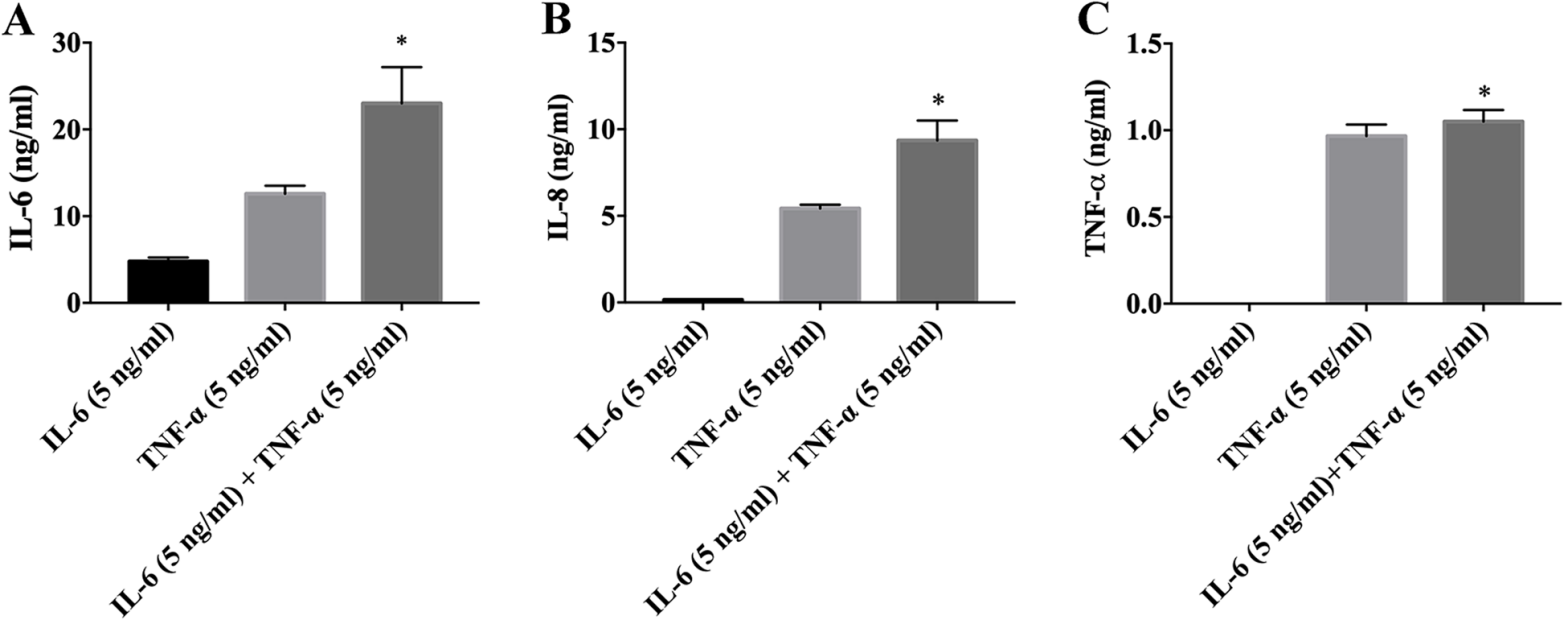
IL-6 and TNF-α-induced pro-inflammatory cytokine expression in ARPE-19 cells. IL-6, TNF-α, and IL-6 + TNF-α at a concentration of 5 ng/ml were used to stimulate ARPE-19 cells for 16 h. Expressions of (**A**) IL-6, (**B**) IL-8, (**C**) TNF-α in ARPE-19 cells were measured using the ELISA Ready-Set-Go kit. The data are expressed as the mean  ± SD of three independent experiments. * *p* < 0.05 compared with the control level

Immunofluorescence results showed increased TNF-α expression levels in RPE [4, 18] of the MFD eye, and that the FJE + PVE treatment resulted in the attenuation of TNF-α levels in the myopic (right) eyes compared to the control group (Right eye MFD) (Supplementary Fig. S1). Therefore, we used human retinal pigment epithelial cell to study the molecular mechanisms on how FJE and PVE lowered the inflammation of the eye. To explore treatment on ARPE-19 cells response of pro-inflammatory cytokines was investigated. ARPE-19 cells were treated with 5 ng/ml of different cytokines, as well as combinations of cytokines (IL-6, TNF-α, and IL-6 + TNF-α), for 16 h. ELISA analysis demonstrated the IL-6 + TNF-α administration significantly increased the levels of IL-6, IL-8, and TNF-α (Fig. 2A-C). IL-6 administration exhibited a lower effect on inflammatory cytokine production. However, when IL-6 and TNF-α were combined, it showed the highest level of inflammatory cytokine production. A synergistic effect was found between IL-6 and TNF-α.

### FJE and FJE + PVE inhibit the inflammatory response in stressed ARPE-19 and human RPE cells

**Fig. 3:**
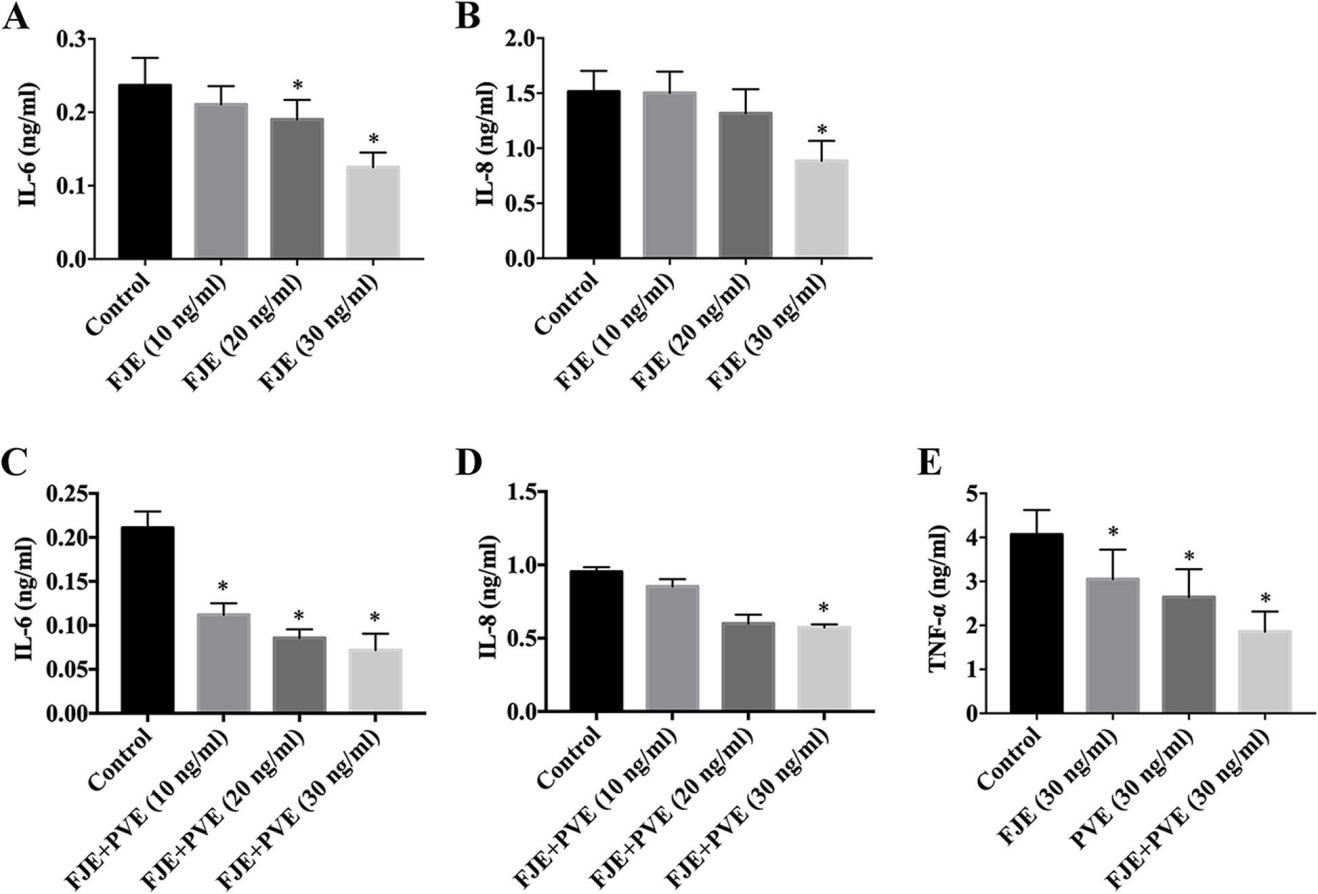
FJE + PVE protects against cytokine-induced inflammation in ARPE-19 cells. ARPE-19 cells were pretreated with 5 ng/ml of IL-6 + TNF-α for 2 h and then FJE was added (10, 20, and 30 ng/ml) and cells were incubated for 6 h. Expressions of IL-6 (**A**) and IL-8 (**B**) in ARPE-19 cells were measured using the ELISA Ready-Set-Go kit. ARPE-19 cells were pretreated with 5 ng/ml of IL-6 + TNF-α for 2 h and then FJE + PVE was added (10, 20, and 30 ng/ml) and the cells were incubated for 6 h. Expression of IL-6 (**C**) and IL-8 (**D**) in ARPE-19 cells were measured using the ELISA Ready-Set-Go kit. ARPE-19 cells were pretreated with 5 ng/ml of IL-6 + TNF-α for 2 h and then 30 ng/ml of FJE, 30 ng/ml of PVE, and 30 ng/ml of FJE + PVE were added and the cells were incubated for 6 h. Expressions of TNF-α (**E**) in ARPE-19 cells were measured using the ELISA Ready-Set-Go kit. The data are expressed as the mean  ± SD of three independent experiments. * *p* < 0.05 compared with the control

**Fig. 4:**
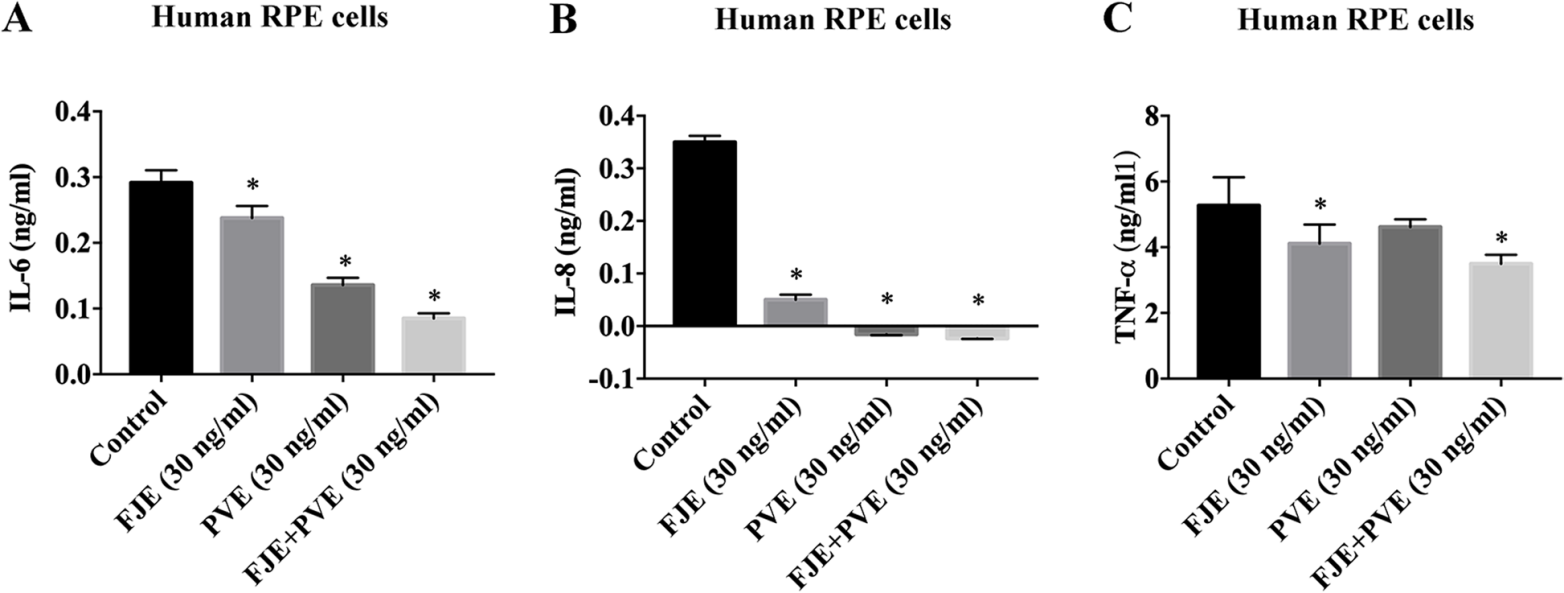
FJE + PVE protects against cytokine-induced inflammation in H-RPE cells. H-RPE cells were pretreated with 5 ng/ml of IL-6 + TNF-α for 2 h and then administered 30 ng/ml FJE, 30 ng/ml PVE, and 30 ng/ml FJE + PVE, and incubated for 6 h. Expressions of IL-6 (**A**), IL-8 (**B**), and TNF-α (**C**) in H-RPE cells were measured using the ELISA Ready-Set-Go kit. The data are expressed as the mean  ± SD of three independent experiments. * *p* < 0.05 compared with the control

The anti-inflammatory effect of FJE was assessed by measuring the production of IL-6 and IL-8. ARPE-19 cells were treated with 5 ng/mL of IL-6 + TNF-α cytokines for 2 h. Treatment media were subsequently removed and fresh media with or without FJE (10, 20, and 30 ng/ml) were applied and incubated for 6 h. When ARPE-19 cells were stimulated with IL-6 + TNF-α, IL-6 and IL-8 production was induced massively. However, cells treated with FJE showed less production of IL-6 and IL-8 compared with those treated with IL-6 + TNF-α, and this inhibitory effect exhibited a dose-dependent manner (Fig. 3A-B). To examine whether the combination of FJE and PVE have additive effects in suppressing IL-6, IL-8, and TNF-α production in IL-6 + TNF-α-stimulated ARPE-19 cells, we applied different concentrations (10, 20, and 30 ng/ml) of FJE + PVE. The levels of IL-6, IL-8, and TNF-α were further reduced when combined treatment of FJE and PVE was applied (Fig. 3C-E). Since IL-6, IL-8, and TNF-α levels were barely detectable when FJE and PVE were combined were at a concentration of over 30 ng/ml, we used a combination of FJE + PVE treatments up to 30 ng/ml in the subsequent experiment. A similar decrease of IL-6, IL-8, and TNF-α levels was also observed in H-RPE cells treated with FJE + PVE (Fig. 4A-C).

### FJE and PVE regulate the phosphorylation of AKT and NF-κB in ARPE-19 cells

**Fig. 5:**
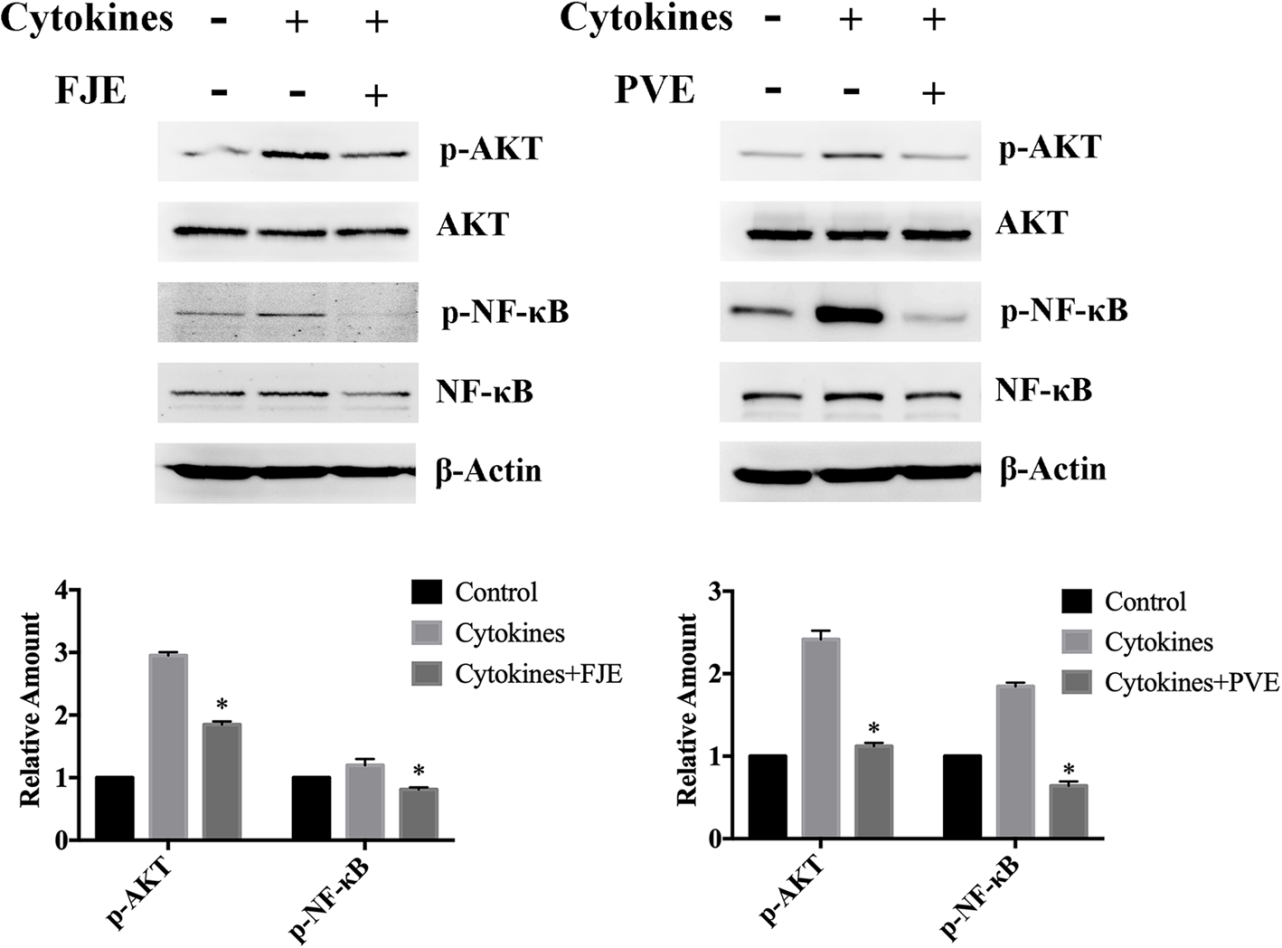
FJE and PVE inhibits cytokine-induced inflammation through attenuating AKT and NF-κB phosphorylation in ARPE-19 cells. ARPE-19 cells were pretreated with 5 ng/ml of IL-6 + TNF-α cytokines for 2 h and then administered FJE and PVE (30 ng/ml) and incubated for 10 min. IL-6 + TNF-α facilitated an increase in AKT and NF-κB phosphorylation levels in treated ARPE-19 cells compared to control cells. β-actin was used as a reference control. Full length blots are presented in Supplementary Fig. S2. Quantification of protein expression levels by normalization to the internal control, β-actin. Depicted western blots are one representative figure of three independent experiments. Results are the mean ± S.D. of three independent experiments

Next, we determined the molecular pathway through which FJE and PVE interfered with the production of pro-inflammatory cytokines by measuring the effects of these compounds on the activation of AKT and NF-κB in IL-6 + TNF-α-treated ARPE-19 cells. ARPE-19 cells were treated with IL-6 + TNF-α, for 2 h. Treatment media were subsequently removed and fresh media with or without FJE and PVE (30 ng/ml) were applied and incubated for 6 h. Results showed that after 6 h of FJE and PVE treatment, phosphorylation levels of AKT and NF-κB decreased markedly compared to cells treated with cytokines (Fig. 5). This result indicated that FJE and PVE inhibited inflammation through downregulation of the AKT and NF-κB pathways.

### FJE, PVE, and FJE + PVE inhibit the progression of myopia

**Fig. 6:**
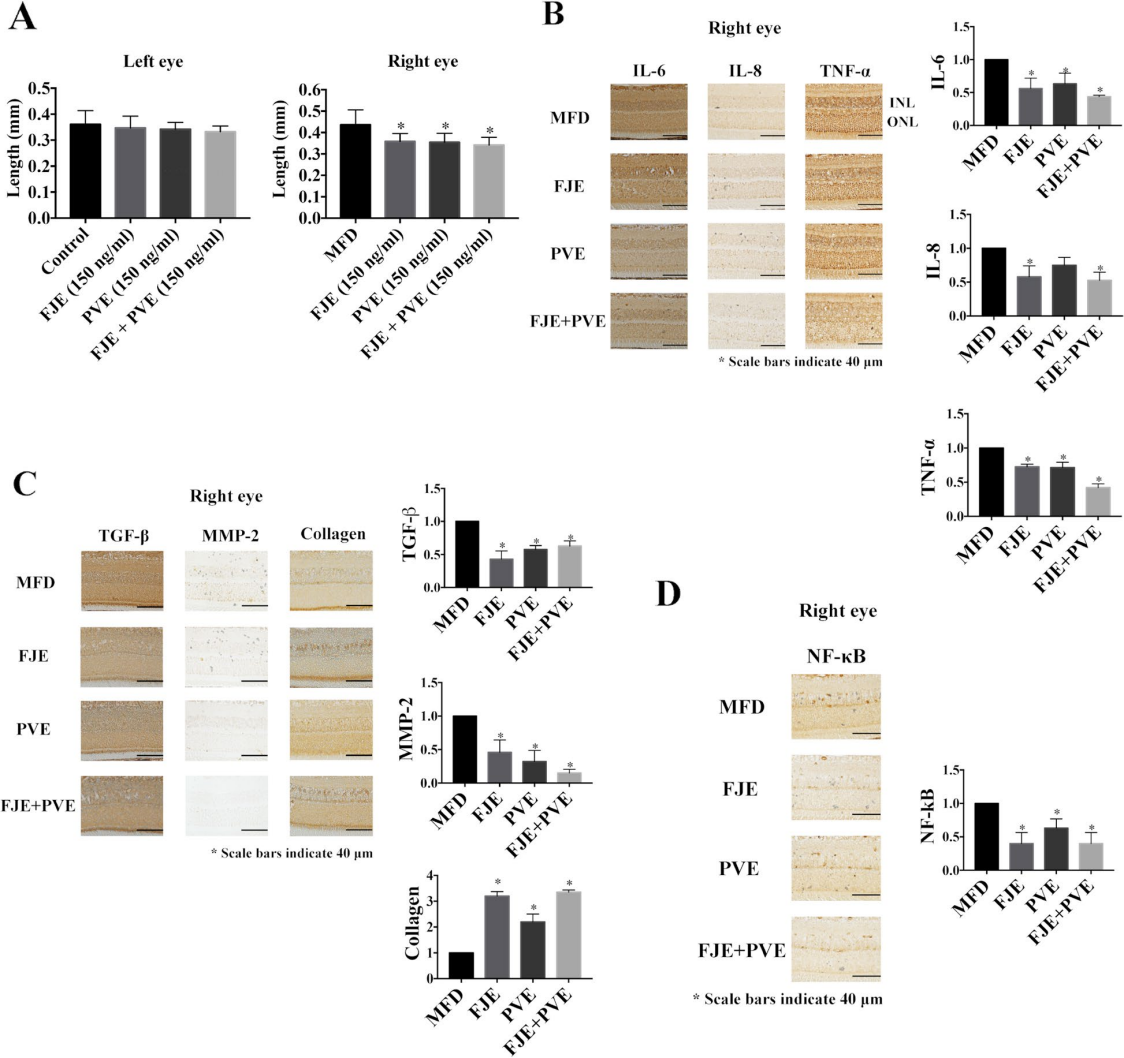
Effect of FJE and PVE on myopia progression and expression levels of inflammation-related proteins in MFD mice. **A** The axial length was determined as the change in axial length measurements before and after MFD for 12 days (*n* = 10 per group). The ANOVA test was used to determine significant differences, and comparisons between control, FJE (150 ng/ml), PVE (150 ng/ml), and FJE + PVE (150 ng/ml). **B** Immunohistochemical analysis of IL-6, IL-8, and TNF-α expression in MFD (Right eye MFD), FJE (150 ng/ml)-treated MFD eyes (Right eye), PVE (150 ng/ml)-treated MFD eyes (Right eye), and FJE + PVE (150 ng/ml)-treated MFD eyes (Right eye). INL: inner nuclear layer; ONL: outer nuclear layer. Quantification of IL-6, IL-8, and TNF-α accumulation in retinas of the right eye using Image J software. **C** Immunohistochemical analysis of TGF-β1, MMP-2, and type I collagen expression in MFD (Right eye control), FJE (150 ng/ml)-treated MFD eyes (Right eye), PVE (150 ng/ml)-treated MFD eyes (Right eye), and FJE + PVE (150 ng/ml)-treated MFD eyes (Right eye). Quantification of TGF-β1, MMP-2, and type I collagen of accumulation in the retinas of the right eye using Image J software. **D** Immunohistochemical analysis of NF-κB expression in MFD (Right eye control), FJE (150 ng/ml)-treated MFD eyes (Right eye), PVE (150 ng/ml)-treated MFD eyes (Right eye), and FJE + PVE (150 ng/ml)-treated MFD eyes (Right eye). Quantification of NF-κB of accumulation in the retinas of the right eye using Image J software. The ANOVA test was used to determine significant differences, and comparisons between control, FJE (150 ng/ml), PVE (150 ng/ml), and FJE + PVE (150 ng/ml). Original IHC are presented in Supplementary Fig. S3. The IHC data are expressed as the mean  ±  SD of three independent experiments. * *p* < 0.05 compared to the control

FJE, PVE and FJE + PVE were applied to MFD hamsters and their axial length was measured 21 days later. Change in the axial length of right eye MFD for the control, FJE- (150 ng/ml), PVE- (150 ng/ml), and FJE + PVE-treated (150 ng/ml) MFD hamsters were 0.442 (mm) ± 0.068, 0.358 (mm) ± 0.037, 0.35 (mm) ± 0.042, and 0.340 (mm) ± 0.036, respectively (*p* < 0.05; Fig. 6A).

### FJE + PVE inhibited myopia progression through modulating the inflammatory response in the eyes

To understand the molecular factors of FJE and PVE on myopia progression, inflammatory proteins were examined in the hamster's eye. As shown in Fig. 6 B, IL-6, IL-8, and TNF-α expressions increased after treatment right eye MFD for 21 days, whereas FJE, PVE, and FJE + PVE treatment reduced their expression. But, the PVE treatment resulted in a no significant (*P* < 0.05) attenuation of IL-8 levels in myopic eyes compared to the MFD group. An elongation of the eye axial length was accompanied with scleral remodeling, such as suppression of collagen production, and TGF-β and MMP-2 activity. Thus, we assessed TGF-β, MMP-2, and collagen expression using IHC (Fig. 6 C). Our results showed that the FJE, PVE, and FJE + PVE treatment resulted in a significant (*P* < 0.05) decrease in TGF-β and MMP-2 expression and an increase in type I collagen expression compared to the MFD group. To further confirm the involvement of inflammatory signaling pathways in myopia progression, the expression of NF-κB was determined using IHC. Expression levels of NF-κB in retinas were lower in FJE-, PVE-, and FJE + PVE-treated eye compared to the MFD groups (Fig. 6 D). Taken together, it was suggested that myopic stimuli induced IL-6, IL-8, TNF-α, TGF-β, MMP-2, and NF-κB overexpression, and decreased collagen expression, and that FJE and PVE reversed these effects.

### Resveratrol + ursolic acid protect against cytokine-induced inflammation in human ARPE-19 and RPE cells

**Fig. 7:**
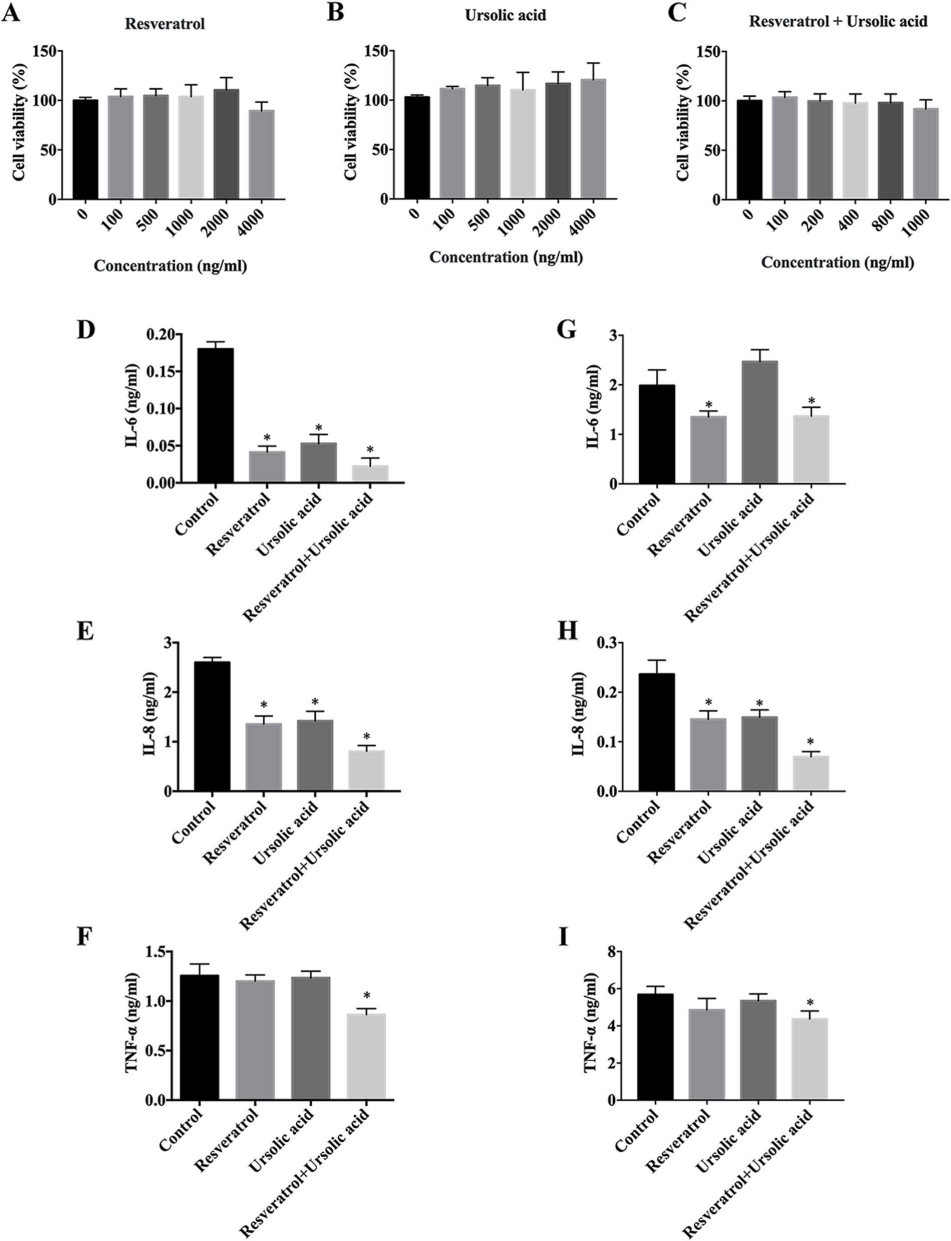
Resveratrol + ursolic acid protect against cytokine-induced inflammation in human ARPE-19 and RPE cells. **A** and **B** Different concentrations of resveratrol and ursolic acid (0, 100, 500, 1000, 2000, and 4000 ng) were applied for 72 h. Cell viability was determined using MTS assay. **C** Different concentrations of resveratrol + ursolic acid (0, 100, 500, 1000, 2000, and 4000 ng/ml) were administered for 72 h. Cell viability was determined using the MTS assay. **D**, **E**, and **F** ARPE-19 cells were pretreated with 5 ng/ml of IL-6 + TNF-α for 2 h and then treated with resveratrol, ursolic acid, and resveratrol + ursolic acid (1000 ng/ml) and incubated for 6 h. Expressions of IL-6, IL-8, and, TNF-α in ARPE-19 cells were measured using the ELISA Ready-Set-Go kit. **G**, **H**, and **I** H-RPE cells were pretreated with 5 ng/ml of IL-6 + TNF-α for 2 h and then administered resveratrol, ursolic acid, and resveratrol + ursolic acid (1000 ng/ml) and incubated for 6 h. Expressions of IL-6, IL-8, and TNF-α in H-RPE cells were measured using the ELISA Ready-Set-Go kit. The data are expressed as the mean  ±  SD of three independent experiments. * *p* < 0.05 compared with the control

To evaluate the cytotoxicity of resveratrol, ursolic acid, and resveratrol + ursolic acid on ARPE-19 cells, the cells were treated with a series of concentrations of resveratrol, ursolic acid, (0, 100, 500, 1000, 2000, and 4000 ng/ml, respectively) and resveratrol + ursolic acid (0, 100, 200, 400, 800, and 1000 ng/ml) for 72 h. As shown in Fig. 7A-C, the viability of ARPE-19 cells was not reduced at resveratrol, ursolic acid, and resveratrol + ursolic acid concentrations lower than 1000 ng/ml. Accordingly, resveratrol, ursolic acid, and resveratrol + ursolic acid concentrations from 1000 ng/ml were chosen for all subsequent experiments. ARPE-19 and H-RPE cells were treated with 5 ng/mL of IL-6 + TNF-α cytokines for 2 h. Treatment media were subsequently removed and fresh media with or without resveratrol, ursolic acid, and resveratrol + ursolic acid (1000 ng/ml) were applied and incubated for 6 h. ELISA results indicated that expression levels of IL-6, IL-8, and TNF-α were significantly increased in IL-6 + TNF-α-stimulated ARPE-19 and H-RPE cells compared with those in untreated cells. Treatment with resveratrol + ursolic acid combination significantly reduced expression levels of IL-6, IL-8, and TNF-α compared to resveratrol and ursolic acid alone. (Fig. 7D-I). These results suggested that resveratrol + ursolic acid could attenuate the production of retinal inflammatory disease-related mediators’ expression levels.

## Discussion

Here, we aimed to investigate the effect of FJ and PV in MFD-induced hamster model and explore the role of inflammatory cytokines in myopia and revealed that FJ and PV extracts attenuated myopia progression via inhibiting inflammation.

In 2020, 3366 million people (42.6% of the population worldwide) had myopia. Patients with myopia carry higher risks of cataract, glaucoma, choroidal neovascularization, and macular and retinal complications, which may result in an irreversible vision loss [19]. Emerging evidence indicated that inflammation has a key role in the pathogenesis of several ocular diseases, including uveitis, age-related macular (AMD), dry eye, and myopia [4, 20]. Inflammation is part of the body’s defense mechanism against infection or injury, which results in cell activation and the release of various mediators responsible for the inflammatory response [21]. In myopia animal model, expressions of IL-6, IL-8, and TNF-α increase when compared with control eyes [4]. IL-6, IL-8, and TNF-α are pro-inflammatory factors involved in the communication between cells and their overexpression has been implicated in the pathogenesis of various inflammatory and ocular diseases [22, 23]. In the present study, we used IL-6, TNF-α or combined with IL-6 and TNF-α to prime the RPE cells, which stimulated the inflammatory signaling molecules in RPE cells. The activation of inflammatory signaling molecules such as NF-κB and Akt will subsequently stimulate the expression of inflammatory cytokines including IL-6, IL-8, and TNF-α. AKT and NF-κB signaling pathways are chose because of their central roles in mediating inflammatory reactions [24, 25]. These inflammatory cytokines will further activate the NF-κB and Akt, which cause a vicious cycle in the RPE layer. The chronic inflammatory reactions in the retina will cause sclera tissue remodeling which lead to axial length elongation and finally myopia. We demonstrated that FJE + PVE suppressed myopia in a hamster MFD model. This was concomitant with the inhibition of MFD-induced increase in IL-6, IL-8, and TNF-α expression, TGF-β, MMP-2, and NF-κB activation, and a decrease in collagen in the MFD hamster model.

Recent studies have revealed the importance of retina, photoreceptors, and retinal pigment epithelium in the regulation of scleral tissue remodeling, by transmitting signals for eye growth and subsequently altering axial length [26]. Stimulating signals that promote scleral tissue remodeling have been shown to derive from the retina and work in conjunction with photoreceptors and the retinal pigment epithelium [27]. The changes in RPE morphology have been recognized in myopia animal models and myopic patients with excessively large eyes [28, 29]. The RPE cells are dead caused by chorioretinal atrophy or expanded resulted from passive stretch promoted by eye enlargement. The enlarged RPE cells was also found in the lid-sutured eyes of a mammalian model [30]. Myopic eyes have the characteristics of a longer axial length, a deeper vitreous chamber, a thinner lens, and a flatter cornea [31, 32]. It is thus suggested that the RPE play a crucial role in eye growth regulation and myopia progression. Moreover, we found a significant increase in the expression of TNF-α in RPE layer in myopic eye compared to control eye (supplementary Fig. 1). We thus use RPE cells to study the molecular mechanisms on how FJE and PVE lowered the inflammatory reactions in the RPE cells.

The present work was undertaken to evaluate in vitro anti-inflammatory activity of FJE, PVE, and FJE + PVE. FJE showed strong anti-inflammatory activity at a concentration of 30 ng/ml. In addition, topical application of FJE + PVE treatment inhibited inflammation to a greater extent when compared to the group treated with either FJE or PVE alone. It has been reported that a high level of cytokines increases the expression of AKT and NF-κB in RPE [33, 34]. Consistent with these reports, results from the present study indicated that cytokine (IL-6 + TNF-α) treatment increased the phosphorylation AKT and NF-κB in ARPE-19 cells. FJE and PVE suppressed IL-6 + TNF-α-induced AKT and NF-κB expression. Therefore, FJE and PVE treatment improved inflammation induced by these cytokines via suppressing AKT and NF-κB signaling pathway.

The use of Chinese herb extracts to treat ophthalmic conditions dates back for hundreds of years. FP and PV are traditional Chinese medicine for the treatment of various inflammatory diseases, like hepatitis and tumors, and are officially listed in the Chinese Pharmacopoeia [35, 36]. In addition, these herbs have anti-inflammatory, anti-oxidant, anti-allergic, anti-bacterial, and anti-viral effects [8, 10, 35].

It has been indicated that compounds with various biological activities are commonly produced by plants. Resveratrol and ursolic acid are the secondary metabolites in FJ and PV and have various pharmacological effects on different diseases [9, 11]. Previous studies reported the anti-inflammatory activity of resveratrol and ursolic acid species [37, 38]. The present study demonstrated that resveratrol + ursolic acid showed the highest anti-inflammatory activity and no cytotoxicity. Collectively, the current data suggested that combined treatment of FJE + PVE and resveratrol + ursolic acid are more effective inhibitors of inflammation than FJE, PVE, resveratrol, or ursolic acid alone. The mechanism for this greater inhibition appeared to be multi-faceted.

Subsequently, we examined the therapeutic effect of FJE, PVE, and FJE + PVE against myopia progression in vivo. Utilizing MFD-induced hamster myopia, we observed that axial elongation changes were suppressed upon FJE, PVE, and FJE + PVE treatments. In our animal models, we demonstrated that FJE + PVE suppressed myopia. This was concomitant with the inhibition of MFD-induced increase in IL-6, IL-8, and TNF-α expressions, TGF-β, MMP-2, and NF-κB activation, and a decrease in collagen expression in the hamster MFD model. Previous studies have shown that expressions of IL-6, IL-8, and TNF-α were higher in uveitis [39]. TNF-α induces the expression of IL-6, and IL-8 expression can be upregulated by TNF-α and IL-6 [40, 41]. In our study, we found that IL-6, IL-8, and TNF-α expressions were higher in the myopia eye. In contrast, IL-6, IL-8, and TNF-α expression decreased in the FJE + PVE-treated group. The development of myopia occurs mainly because of excessive axial length rather than changes in cornea or lens power. In animal models of myopia, there is a loss of extracellular matrix (ECM), which may cause axial elongation. Induction of myopia leads to increased TGF-β expression and continues to activate MMP-2 expression. MMP-2 is an enzyme that cleaves collagen I and capable of triggering the decomposition of scleral ECM components. Furthermore, in hamsters with myopia, an increased expression of MMP-2 was induced by TGF-β through NF-κB activation. In this study, we demonstrated that while MFD enhanced TGF-β and MMP-2 activity, FJE + PVE reversed this effect.

## Conclusions

**Fig. 8:**
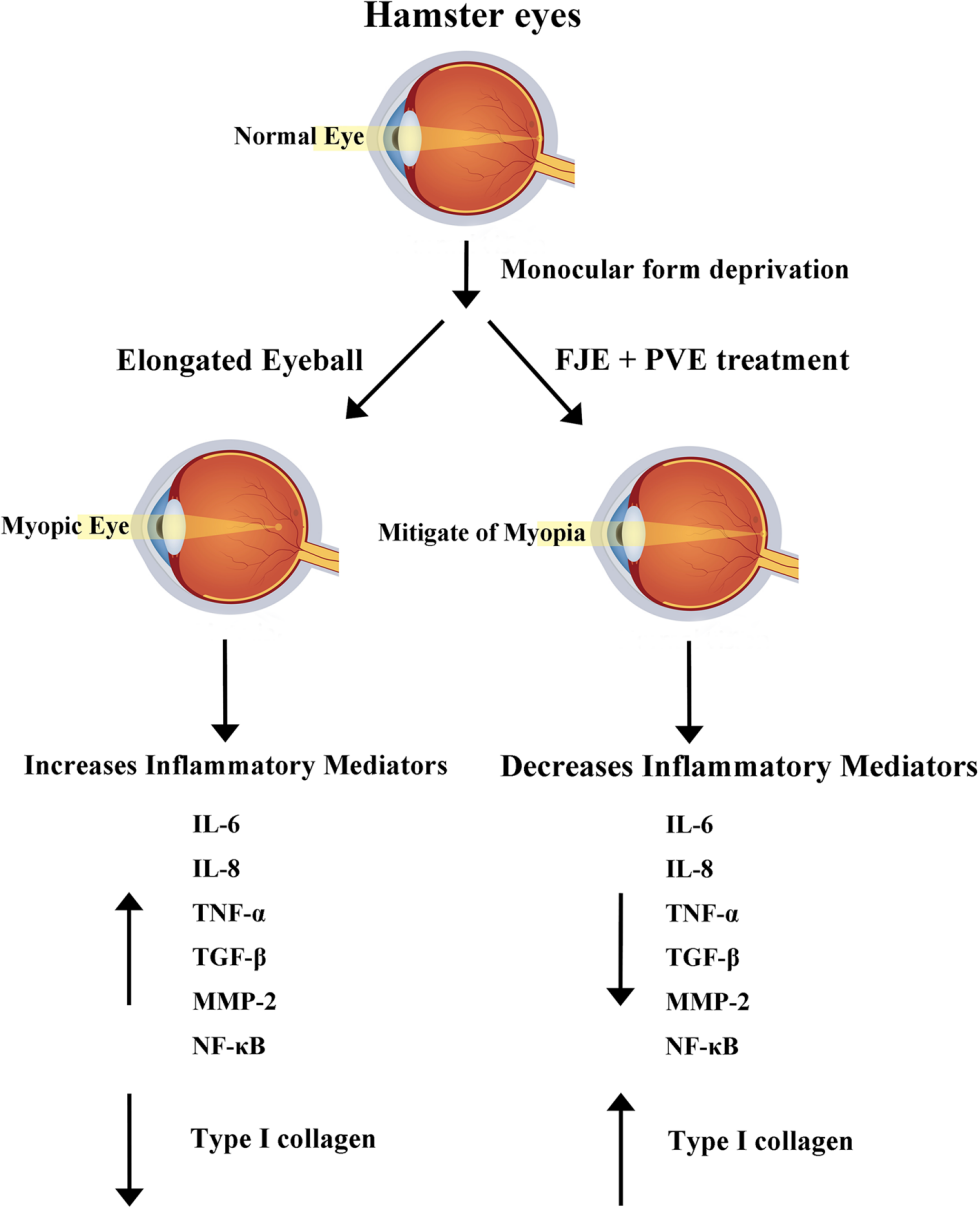
Schematic presentation of how effect of combined treatment with *Fallopia Japonica* and *Prunella vulgaris* extract on myopia progress

In conclusion, the current study shows the efficacy of the combination of FJE + PVE in the inhibition of myopia progression in hamster eyes for the first time (Fig. 8). This combination resulted in greater inhibition of myopia progression compared to FJE or PVE treatment alone. In addition, resveratrol and ursolic acid are the secondary metabolites in FJ and PV and they also have inhibitory effects on inflammation. Emerging evidence suggests that combinations of phytochemicals may have more effective anti-inflammatory effects than single agents [38]. Hence, FJE + PVE are considered beneficial to prevent myopia development in humans.

## Supplementary Information

**Additional file 1.**

## Data Availability

The datasets generated and analyzed during the current study are available from the corresponding author on reasonable request.

## Abbreviations

MTS: 3-(4,5-Dimethylthiazol-2-yl)-5-(3-carboxymethoxyphenyl)-2(4-sulfophenyl)-2H-tetrazolium, inner salt)
AMD: Age-related macular degeneration
DMEM: Dulbecco’ Modified Eagle’s medium
FJ: *Fallopia Japonica*
FJE: *Fallopia Japonica* Extract
FBS: Fetal bovine serum
H-RPE: Human RPE
IHC: Immunohistochemistry
IL: Interleukin
MMP: Matrix metalloproteinase
MFD: Monocular form deprivation
NF-κB: Nuclear factor kappa-light-chain-enhancer of activated B
PS: Penicillin–streptomycin
PMS: Phenazine methosulfate
AKT: Protein kinase B
PV: *Prunella Vulgaris*
PVE: *Prunella Vulgaris* Extract
RPE: Retinal pigment epithelium
SD: Standard deviation
TGF-β: Transforming growth factor beta

## References

[1] Holden BA, Fricke TR, Wilson DA, Jong M, Naidoo KS, Sankaridurg P, Wong TY, Naduvilath TJ, Resnikoff S (2016). Global prevalence of myopia and high myopia and temporal trends from 2000 through 2050. Ophthalmology.

[2] Williams K, Hammond C (2019). High myopia and its risks. Community Eye Health.

[3] Rymer J, Wildsoet CF (2005). The role of the retinal pigment epithelium in eye growth regulation and myopia: a review. Vis Neurosci.

[4] Lin HJ, Wei CC, Chang CY, Chen TH, Hsu YA, Hsieh YC, Chen HJ, Wan L (2016). Role of chronic inflammation in myopia progression: clinical evidence and experimental validation. EBioMedicine.

[5] Kung YJ, Wei CC, Chen LA, Chen JY, Chang CY, Lin CJ, Lim YP, Tien PT, Chen HJ, Huang YS (2017). Kawasaki disease increases the incidence of Myopia. Biomed Res Int.

[6] Wei CC, Kung YJ, Chen CS, Chang CY, Lin CJ, Tien PT, Chang HY, Chen HJ, Huang YS, Lin HJ (2018). Allergic conjunctivitis-induced retinal inflammation promotes myopia progression. EBioMedicine.

[7] Yuan J, Wu S, Wang Y, Pan S, Wang P, Cheng L (2019). Inflammatory cytokines in highly myopic eyes. Sci Rep.

[8] Eid SY, El-Readi MZ, Ashour ML, Wink M (2015). Fallopia japonica, a natural modulator, can overcome multidrug resistance in cancer cells. Evid Based Complement Alternat Med.

[9] Lachowicz S, Oszmianski J. Profile of Bioactive Compounds in the Morphological Parts of Wild Fallopia japonica (Houtt) and Fallopia sachalinensis (F. Schmidt) and Their Antioxidative Activity. Molecules. 2019;24(7):1436. 10.3390/molecules24071436 PMC6479739 30979044

[10] Wang SJ, Wang XH, Dai YY, Ma MH, Rahman K, Nian H, Zhang H (2019). Prunella vulgaris: a comprehensive review of chemical constituents, pharmacological effects and clinical applications. Curr Pharm Des.

[11] Li BY, Hu Y, Li J, Shi K, Shen YF, Zhu B, Wang GX (2019). Ursolic acid from Prunella vulgaris L. efficiently inhibits IHNV infection in vitro and in vivo. Virus Res.

[12] Chen H, Tuck T, Ji X, Zhou X, Kelly G, Cuerrier A, Zhang J (2013). Quality assessment of Japanese knotweed (Fallopia japonica) grown on Prince Edward Island as a source of resveratrol. J Agric Food Chem.

[13] Qiang Z, Ye Z, Hauck C, Murphy PA, McCoy JA, Widrlechner MP, Reddy MB, Hendrich S (2011). Permeability of rosmarinic acid in Prunella vulgaris and ursolic acid in Salvia officinalis extracts across Caco-2 cell monolayers. J Ethnopharmacol.

[14] Ryu SY, Oak MH, Yoon SK, Cho DI, Yoo GS, Kim TS, Kim KM (2000). Anti-allergic and anti-inflammatory triterpenes from the herb of Prunella vulgaris. Planta Med.

[15] Fujikado T, Kawasaki Y, Suzuki A, Ohmi G, Tano Y (1997). Retinal function with lens-induced myopia compared with form-deprivation myopia in chicks. Graefes Arch Clin Exp Ophthalmol.

[16] Thomson K, Karouta C, Ashby R (2020). Form-deprivation and lens-induced myopia are similarly affected by pharmacological manipulation of the dopaminergic system in chicks. Invest Ophthalmol Vis Sci.

[17] Ikeda SI, Kurihara T, Toda M, Jiang X, Torii H, Tsubota K (2020). Oral Bovine Milk Lactoferrin Administration Suppressed Myopia Development through Matrix Metalloproteinase 2 in a Mouse Model. Nutrients..

[18] Marchese A, Carnevali A, Sacconi R, Centoducati T, Querques L, Bandello F, Querques G (2017). Retinal Pigment Epithelium Humps in High Myopia. Am J Ophthalmol.

[19] Theophanous C, Modjtahedi BS, Batech M, Marlin DS, Luong TQ, Fong DS (2018). Myopia prevalence and risk factors in children. Clin Ophthalmol.

[20] Bose T, Diedrichs-Mohring M, Wildner G (2016). Dry eye disease and uveitis: a closer look at immune mechanisms in animal models of two ocular autoimmune diseases. Autoimmun Rev.

[21] Schmid-Schonbein GW (2006). Analysis of inflammation. Annu Rev Biomed Eng.

[22] Massingale ML, Li X, Vallabhajosyula M, Chen D, Wei Y, Asbell PA (2009). Analysis of inflammatory cytokines in the tears of dry eye patients. Cornea.

[23] Ulhaq ZS, Soraya GV, Budu, Wulandari LR (2020). The role of IL-6-174 G/C polymorphism and intraocular IL-6 levels in the pathogenesis of ocular diseases: a systematic review and meta-analysis. Sci Rep.

[24] Dhapola R, Hota SS, Sarma P, Bhattacharyya A, Medhi B, Reddy DH (2021). Recent advances in molecular pathways and therapeutic implications targeting neuroinflammation for Alzheimer's disease. Inflammopharmacology.

[25] Sun Q, Gong T, Liu M, Ren S, Yang H, Zeng S, Zhao H, Chen L, Ming T, Meng X (2022). Shikonin, a naphthalene ingredient: Therapeutic actions, pharmacokinetics, toxicology, clinical trials and pharmaceutical researches. Phytomedicine.

[26] McBrien NA, Lawlor P, Gentle A (2000). Scleral remodeling during the development of and recovery from axial myopia in the tree shrew. Invest Ophthalmol Vis Sci.

[27] Chen KC, Hsi E, Hu CY, Chou WW, Liang CL, Juo SH (2012). MicroRNA-328 may influence myopia development by mediating the PAX6 gene. Invest Ophthalmol Vis Sci.

[28] Ohno-Matsui K, Lai TY, Lai CC, Cheung CM (2016). Updates of pathologic myopia. Prog Retin Eye Res.

[29] Lin T, Grimes PA, Stone RA (1993). Expansion of the retinal pigment epithelium in experimental myopia. Vision Res.

[30] Zhang Y, Wildsoet CF (2015). RPE and choroid mechanisms underlying ocular growth and Myopia. Prog Mol Biol Transl Sci.

[31] Gwiazda J, Hyman L, Hussein M, Everett D, Norton TT, Kurtz D, Leske MC, Manny R, Marsh-Tootle W, Scheiman M (2003). A randomized clinical trial of progressive addition lenses versus single vision lenses on the progression of myopia in children. Invest Ophthalmol Vis Sci.

[32] Zadnik K, Mutti DO, Friedman NE, Qualley PA, Jones LA, Qui P, Kim HS, Hsu JC, Moeschberger ML (1999). Ocular predictors of the onset of juvenile myopia. Invest Ophthalmol Vis Sci.

[33] Gong C, Qiao L, Feng R, Xu Q, Zhang Y, Fang Z, Shen J, Li S (2020). IL-6-induced acetylation of E2F1 aggravates oxidative damage of retinal pigment epithelial cell line. Exp Eye Res.

[34] Chen X, Han R, Hao P, Wang L, Liu M, Jin M, Kong D, Li X (2018). Nepetin inhibits IL-1beta induced inflammation via NF-kappaB and MAPKs signaling pathways in ARPE-19 cells. Biomed Pharmacother.

[35] Fan P, Zhang T, Hostettmann K (2013). Anti-inflammatory Activity of the Invasive Neophyte Polygonum Cuspidatum Sieb. and Zucc. (Polygonaceae) and the Chemical Comparison of the Invasive and Native Varieties with regard to Resveratrol. J Tradit Complement Med.

[36] Hwang YJ, Lee EJ, Kim HR, Hwang KA (2013). NF-kappaB-targeted anti-inflammatory activity of Prunella vulgaris var. lilacina in macrophages RAW 264.7. Int J Mol Sci.

[37] Ding YJ, Sun CY, Wen CC, Chen YH (2015). Nephroprotective role of resveratrol and ursolic Acid in aristolochic Acid intoxicated zebrafish. Toxins (Basel).

[38] Cho J, Rho O, Junco J, Carbajal S, Siegel D, Slaga TJ, DiGiovanni J (2015). Effect of combined treatment with ursolic acid and resveratrol on skin tumor promotion by 12-O-Tetradecanoylphorbol-13-Acetate. Cancer Prev Res (Phila).

[39] Zelazowska-Rutkowska B, Mrugacz M, Cylwik B (2017). Comparison of the Diagnostic Power of Serum IL-6, IL-8 and TNF-alpha for the Idiopathic Anterior Uveitis in Children. Clin Lab.

[40] Kurokouchi K, Kambe F, Yasukawa K, Izumi R, Ishiguro N, Iwata H, Seo H (1998). TNF-alpha increases expression of IL-6 and ICAM-1 genes through activation of NF-kappaB in osteoblast-like ROS17/2.8 cells. J Bone Miner Res.

[41] Xie K (2001). Interleukin-8 and human cancer biology. Cytokine Growth Factor Rev.

[42] Lin CH, Chen CS, Wang YC, Lin ES, Chang CY, Chen JY, Wu MY, Lin HJ, Wan L (2021). Fallopia Japonica and Prunella Vulgaris Inhibit Myopia Progression by Suppressing Akt and NFκB Mediated Inflammatory Reactions. Preprints.

