# Supplementary material for: Fallopia Japonica and Prunella vulgaris inhibit myopia progression by suppressing AKT and NFκB mediated inflammatory reactions
Source: BMC Complement Med Ther. 2022 Oct 14;22:271. doi: 10.1186/s12906-022-03747-2 (PMC9563826; doi:10.1186/s12906-022-03747-2)

**Supplementary information**

Chih-Sheng Chen^1,2^, Yu-An Hsu^3^, Chia-Hung Lin^3^, Yao-Chien Wang^4^, En-Shyh Lin^5^, Ching-Yao Chang^6^, Jamie Jiin-Yi Chen^7^, Ming-Yen Wu^7^, Hui-Ju Lin^3,7,*^,

Lei Wan^3,6,8,*^

1. Department of Food Nutrition and Health Biotechnology, Asia University, Taichung, Taiwan
2. Division of Chinese Medicine, Asia University Hospital, Taichung, Taiwan
3. School of Chinese Medicine, China Medical University, Taichung, Taiwan
4. Department of Emergency Medicine, Taichung Tzu Chi Hospital, Taichung, Taiwan
5. Department of Beauty Science, National Taichung University of Science and Technology, Taichung, Taiwan
6. Department of Medical Laboratory Science and Biotechnology, Asia University, Taichung, Taiwan
7. Eye center, China Medical University Hospital, Taichung, Taiwan
8. Department of Obstetrics and Gynecology, China Medical University Hospital, Taichung, Taiwan

Figure of Contents

**Supplementary Fig. S1**

**Supplementary Fig. S2**

**Supplementary Fig. S3**

**Supplementary data**

**Supplementary Fig. S1**

**
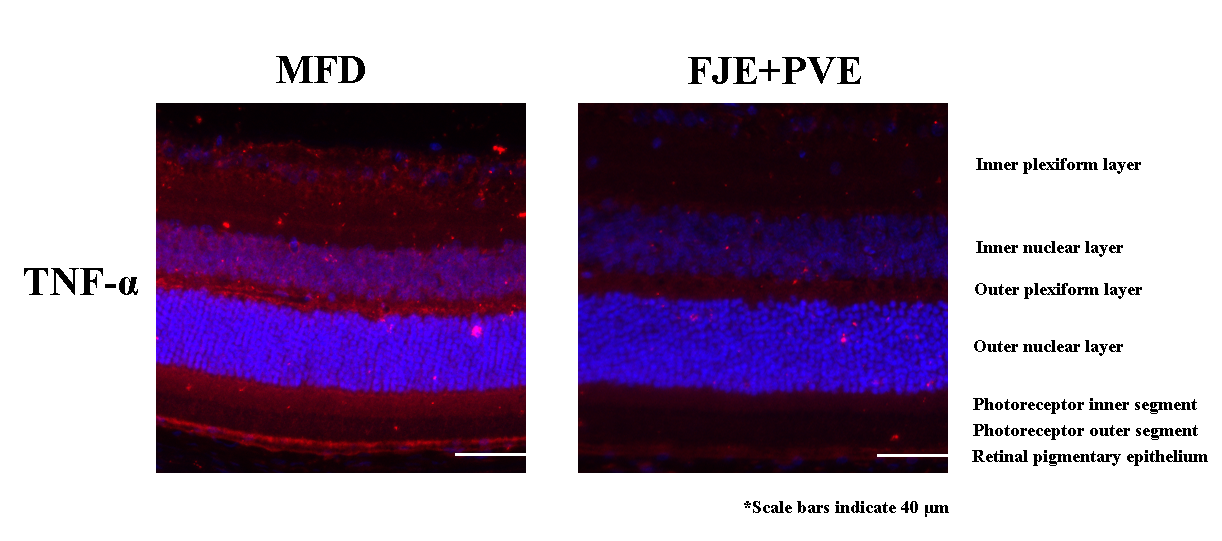
**

**Supplementary Fig. S1: Increased expression of TNF-α in retina of hamsters after MFD induced myopia progression**

Immunofluorescence analysis of TNF-α expression in MFD eyes (Right eye MFD), and FJE + PVE (150 ng/ml)-treated MFD eyes (Right eye).

**Supplementary Fig. S2: Original Blots of Fig. 5**

**
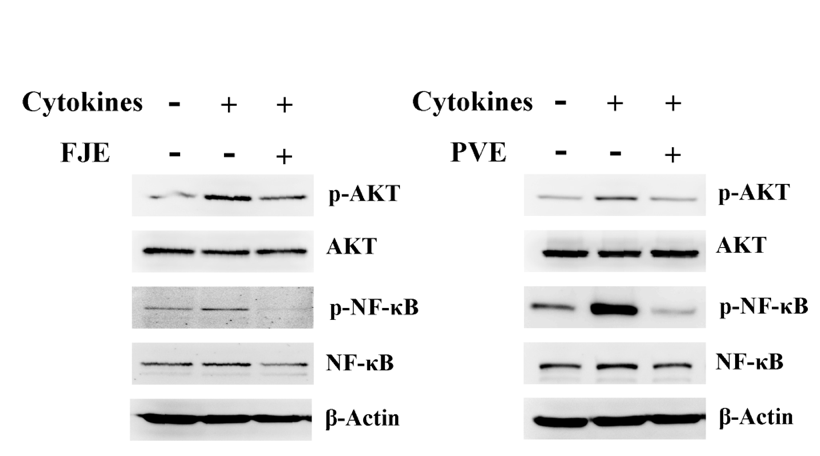
**

**Uncropped western blot images**


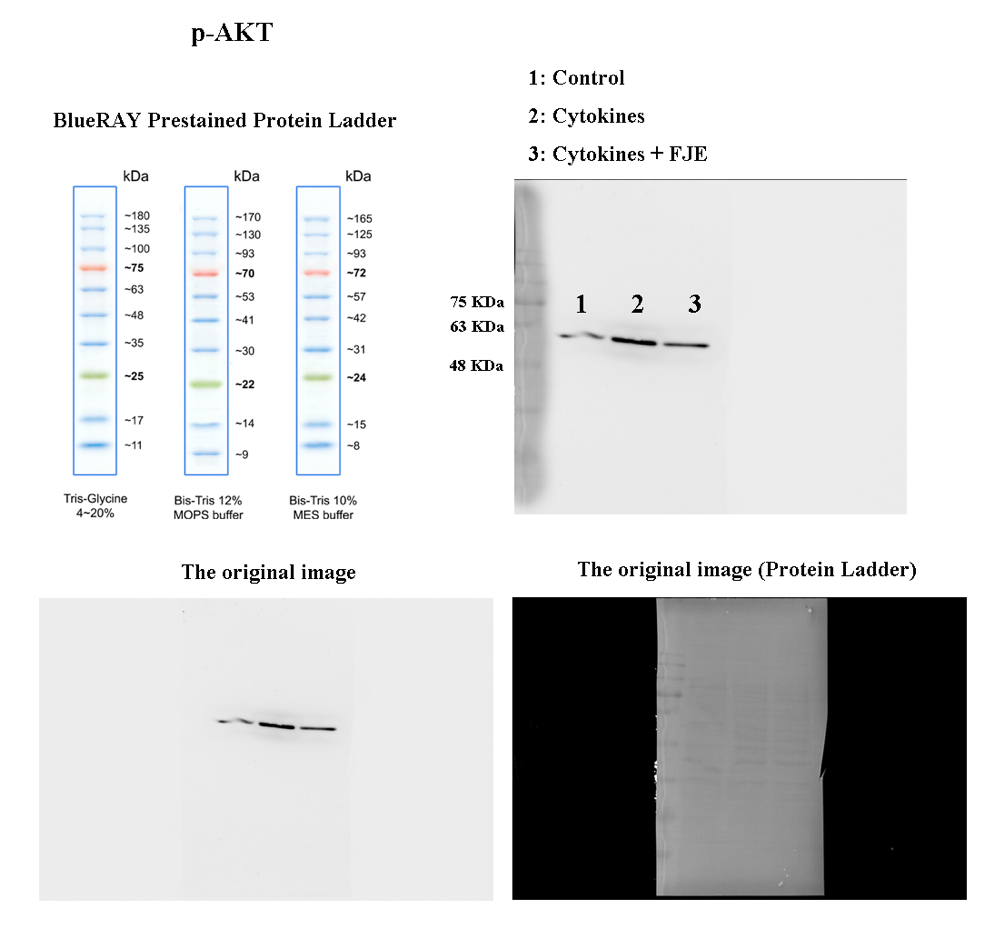


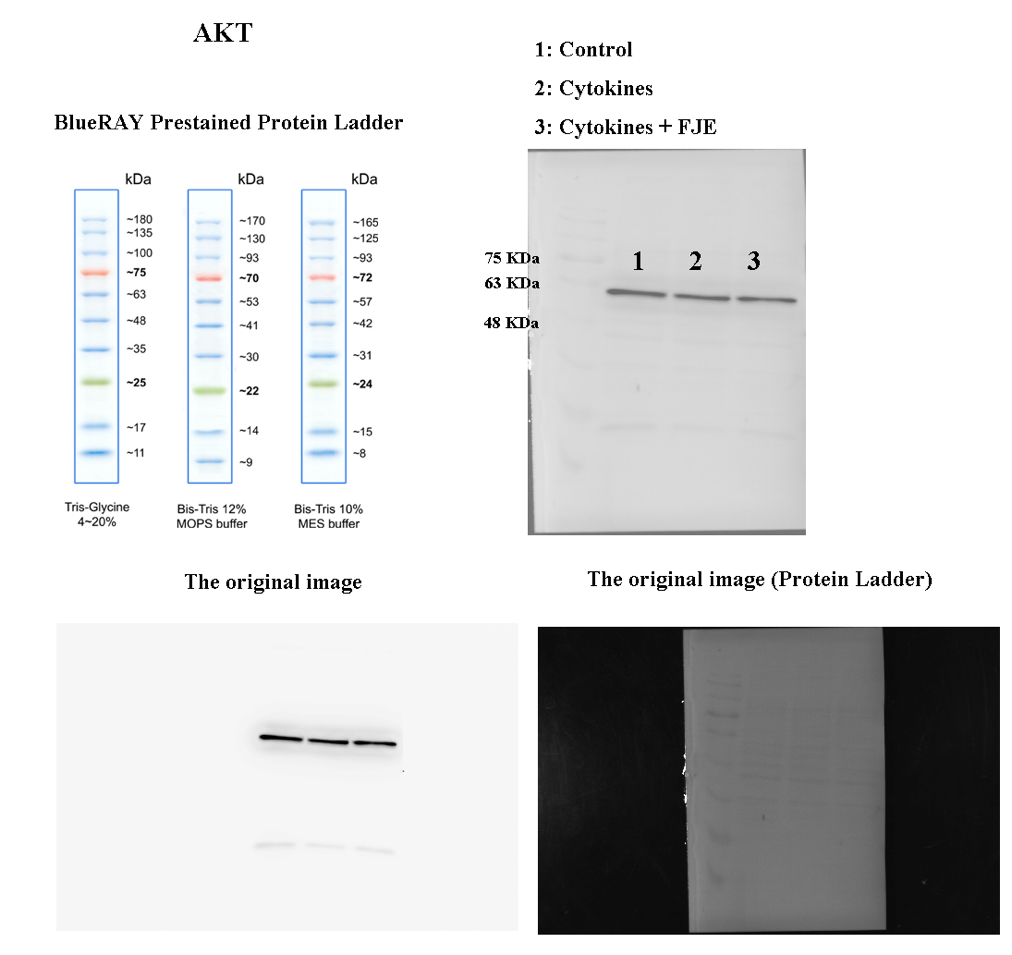


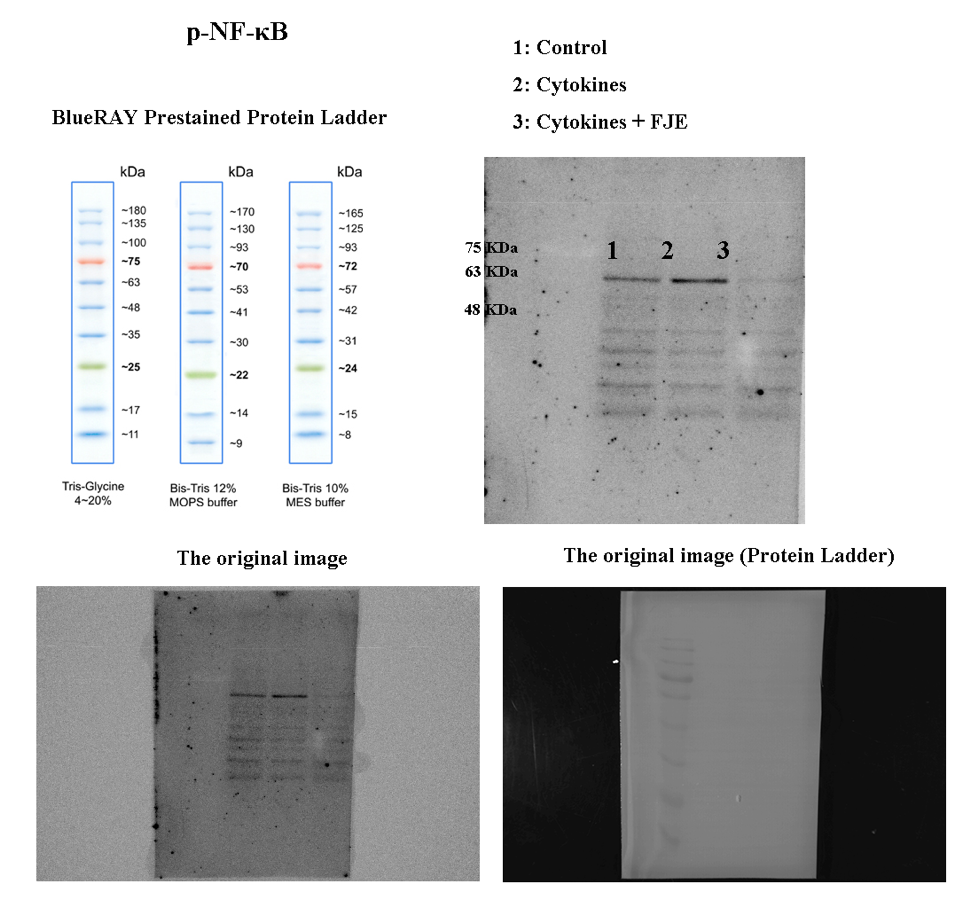


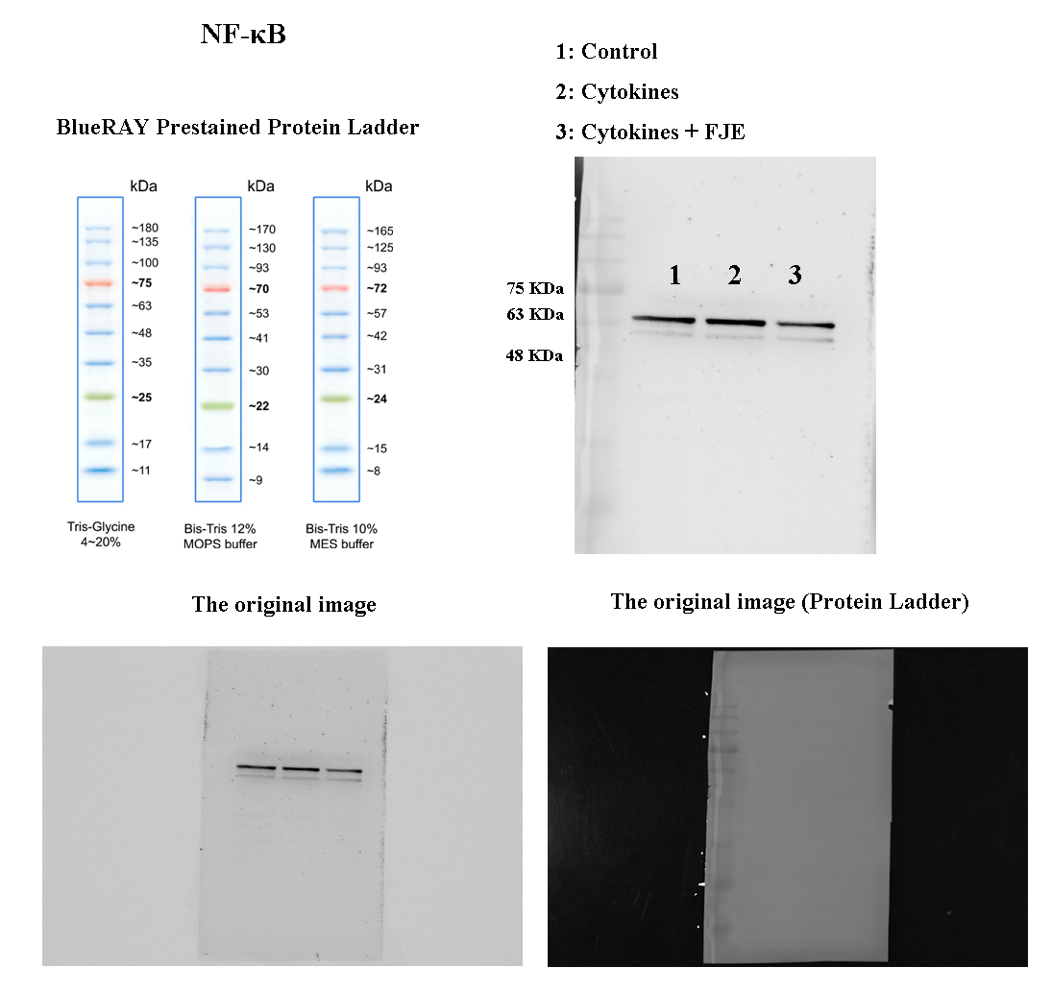


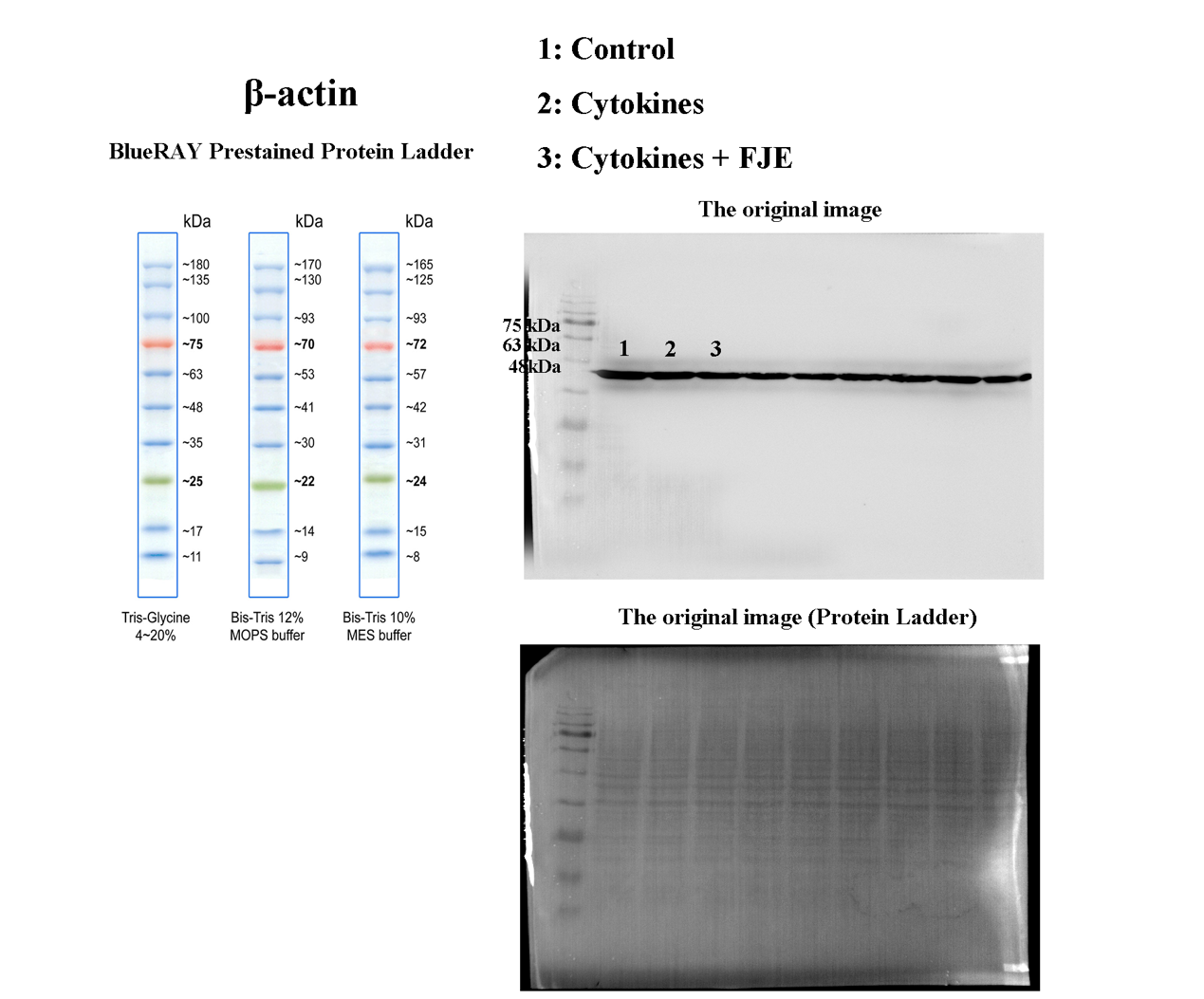


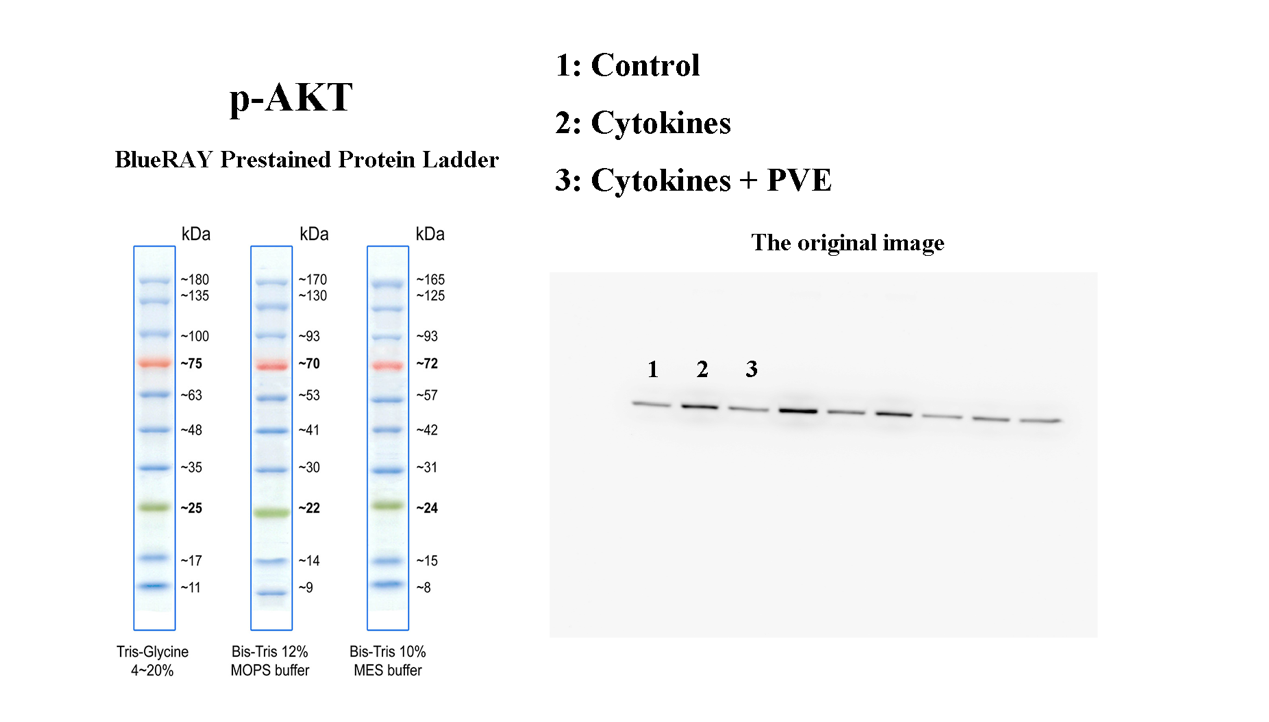


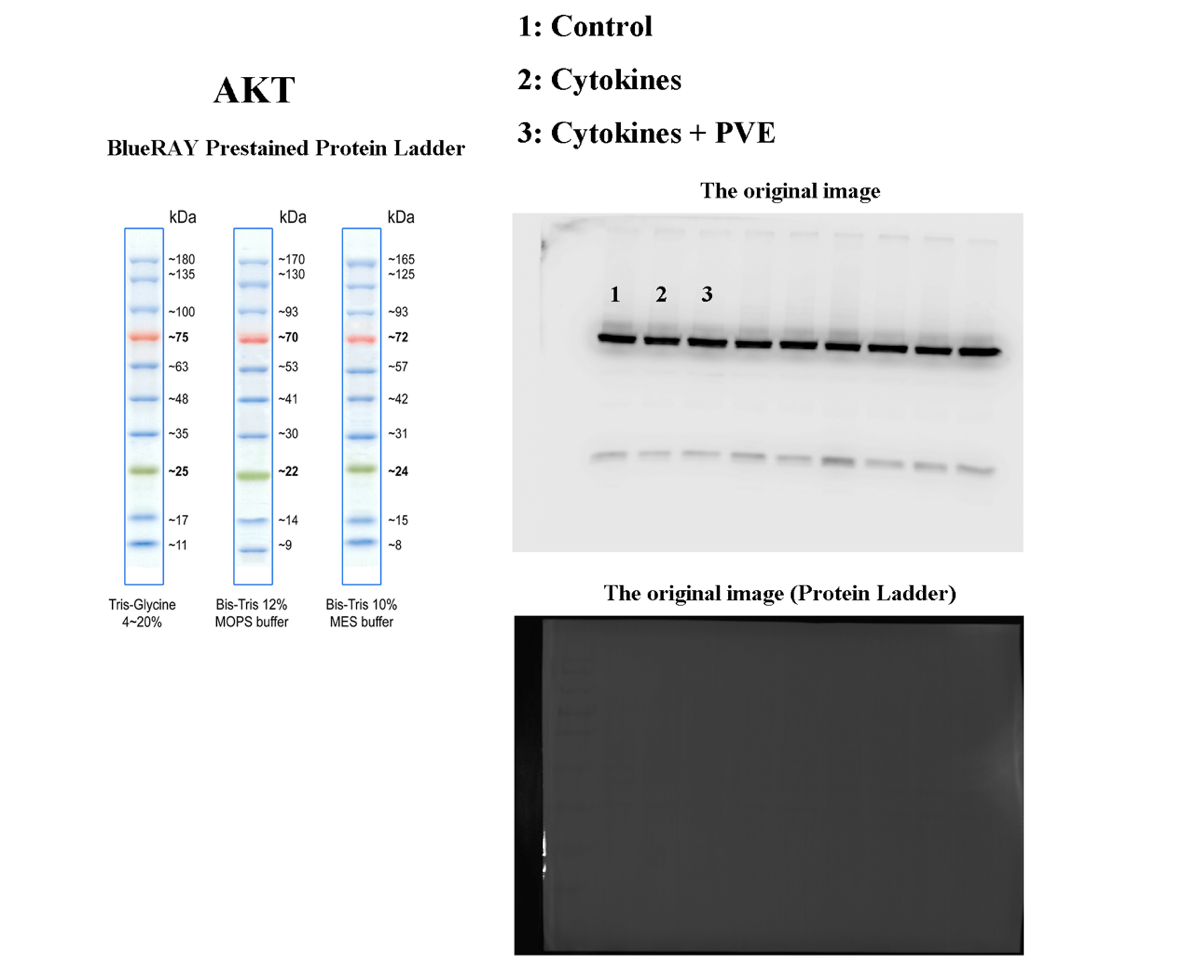


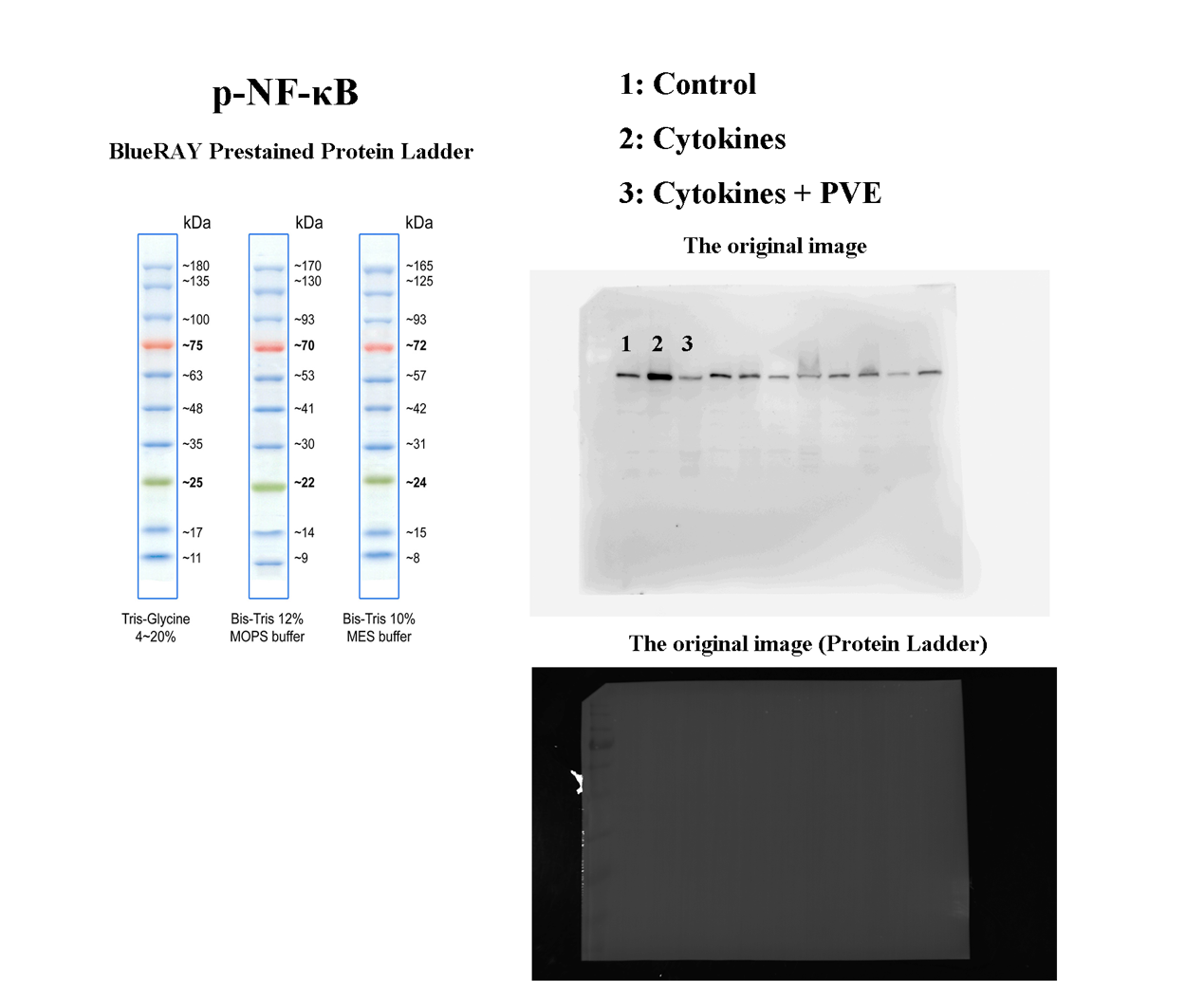


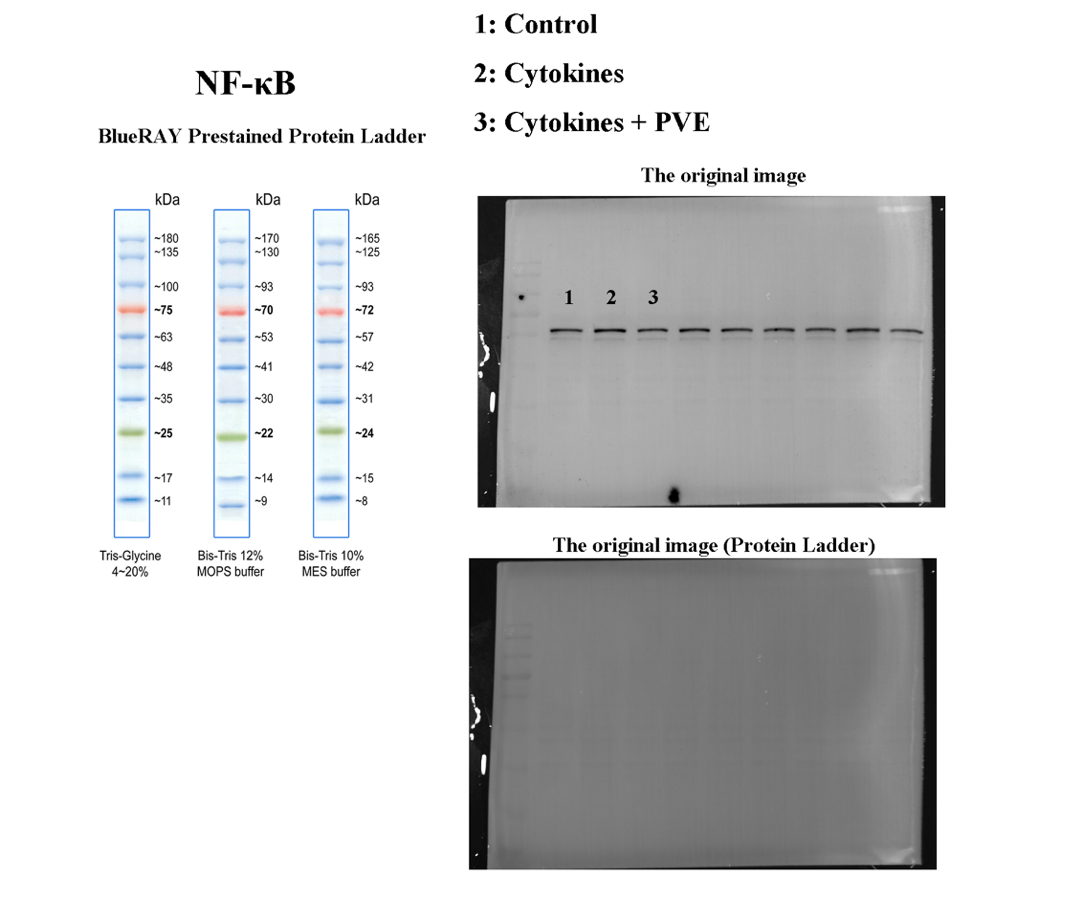


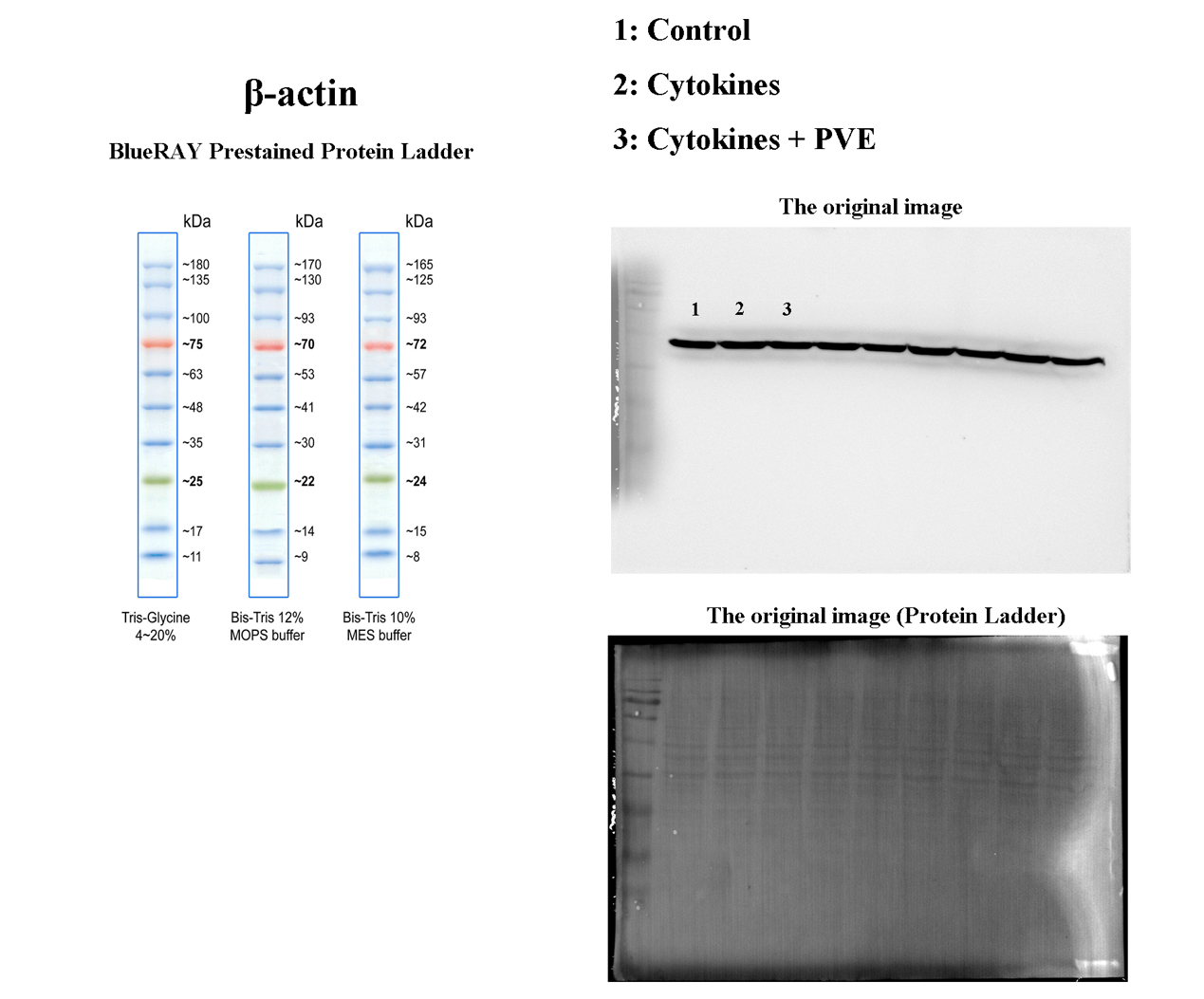


**Supplementary Fig. S3: Original IHC of Fig. 6**

**Uncropped IHC images**

**B**


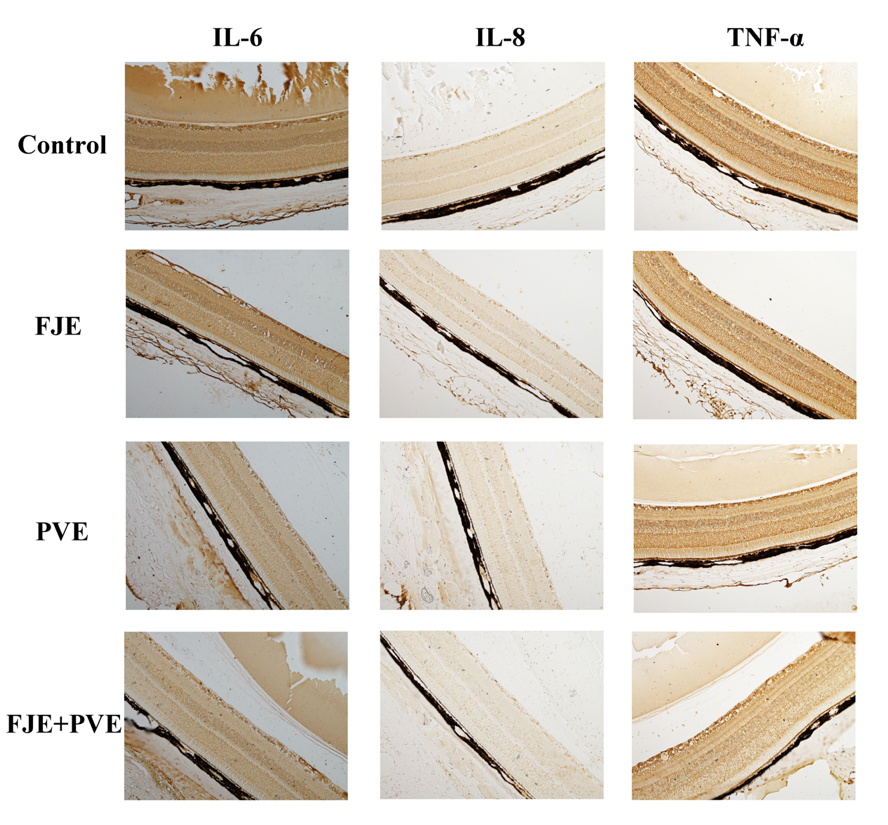


C


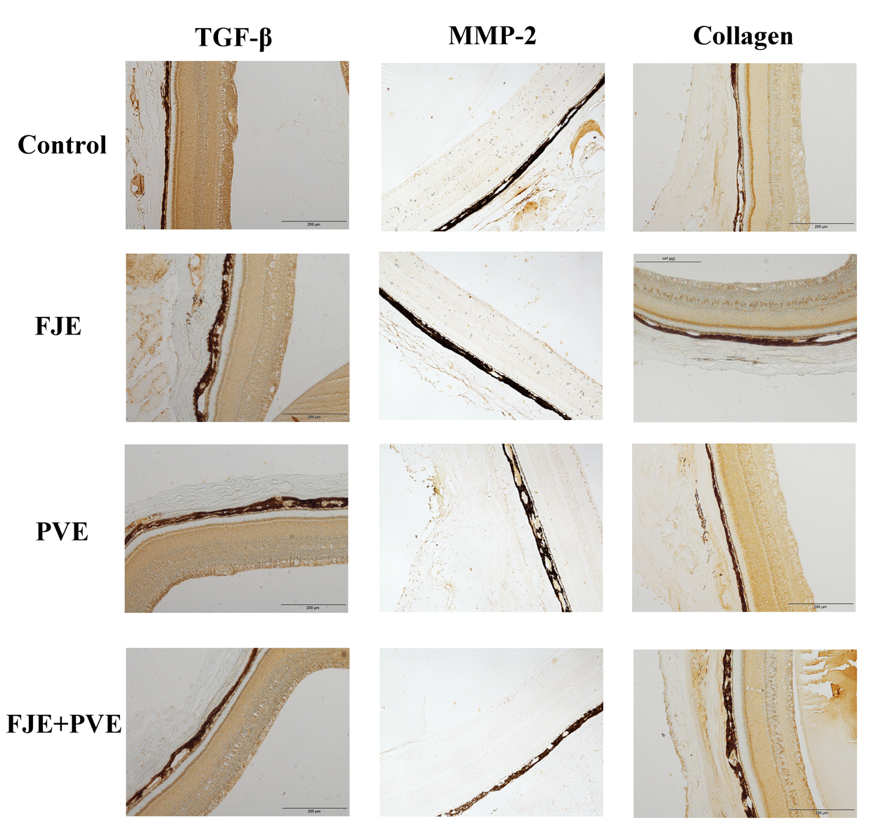


D


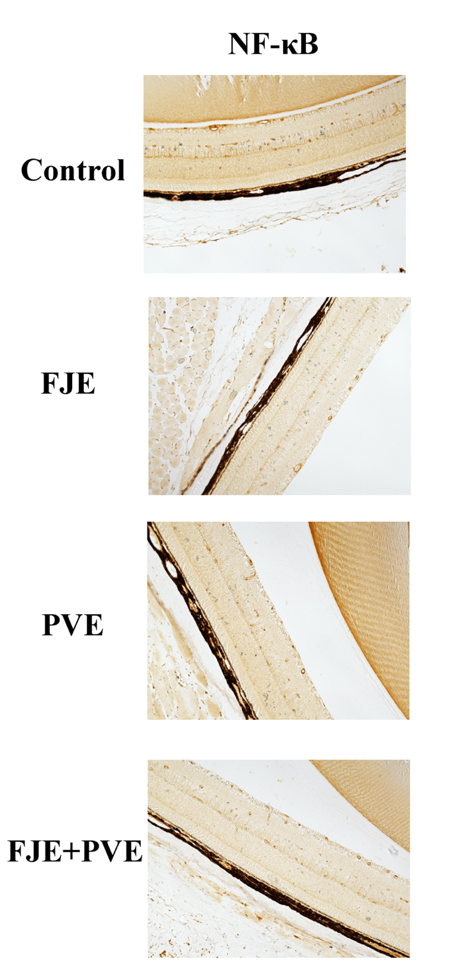

Supplement: Supplementary file 1 — Additional file 1. [file 12906_2022_3747_MOESM1_ESM.docx]
